# Long-Range Ruthenium-Amine Electronic Communication through the *para*-Oligophenylene Wire

**DOI:** 10.1038/srep13835

**Published:** 2015-09-07

**Authors:** Jun-Jian Shen, Yu-Wu Zhong

**Affiliations:** 1 Beijing National Laboratory for Molecular Sciences, CAS Key Laboratory of Photochemistry, Institute of Chemistry, Chinese Academy of Sciences, Beijing 100190, China

## Abstract

The studies of long-range electronic communication are hampered by solubility and potential-splitting issues. A “hybridized redox-asymmetry” method using a combination of organic and inorganic redox species is proposed and exemplified to overcome these two issues. Complexes **1**(PF_6_)–**6**(PF_6_) (from short to long in length) with the organic redox-active amine and inorganic cyclometalated ruthenium termini bridged by the para-oligophenylene wire have been prepared. Complex **6** has the longest Ru-amine geometrical distance of 27.85 Å. Complexes **3**(PF_6_) and **4**(PF_6_) show lamellar crystal packing on the basis of a head-to-tail anti-parallelly aligned dimeric structure. Two redox waves are observed for all complexes in the potential region between +0.2 and +0.9 V vs Ag/AgCl. The electrochemical potential splitting is 410, 220, 143, 112, 107, and 105 mV for **1**(PF_6_) through **6**(PF_6_), respectively. Ruthenium (+2) to aminium (N^•+^) charge transfer transitions have been identified for the odd-electron compounds **1**^2+^–**6**^2+^ by spectroelectrochemical measurements. The electronic communication between amine and ruthenium decreases exponentially with a decay slope of −0.137 Å^−1^. DFT calculations have been performed to complement these experimental results.

The study of molecular wire-mediated electron/energy transfer between electron donor and acceptor is of great significance for molecular electronics^1^,^2^,^3^,^4^,^5^. *para*-Oligophenylene is one of the important molecular wires that have received much attention in photoinduced electron/energy transfer processes^6^,^7^,^8^,^9^,^10^. These processes occur primarily via coherent superexchange tunneling, though incoherent hopping may take place in the long-range regime^9^,^10^.

The fundamental photoinduced electron transfer process has been investigated for many years, including those studied in mixed valency chemistry as pioneered by Creutz and Taube^11^,^12^,^13^,^14^,^15^. However, it remains a challenge to examine the degree of electronic communication between redox sites separated by a long molecular wire^16^,^17^,^18^. The realization and quantification of electronic communication over a distance of 25 Å is very difficult and known examples are very rare. Launay and Collin and co-workers reported the presence of electronic communication across a 24 Å metal-metal distance in 1998^19^. Recently, the ruthenium-ruthenium electronic coupling through a polyyn-diyl bridge with a distance of 25.5 Å has been reported by Ren and Crutchley and coworkers^20^. When the bridge distance was further elongated to 28 Å, no electronic coupling has been evidenced.

We consider that the studies of long-range electronic communication in mixed-valency chemistry are hampered by two important issues: the solubility and potential-splitting issues. Taking the *para*-oligophenylene wire as an example, it is well known that the solubility of *para*-oligophenylene derivatives decrease significantly with increasing length (the solubility issue)^21^,^22^. Compounds with more than five repeating phenyl units and no solubilizing side chains are very limited^22^. The presence of solubilizing side chains is problematic because they cause an increased phenyl-phenyl twist and hence decoupling of the repeating units. For mixed-valent system with strong coupling, a large electrochemical potential splitting Δ*E* is often observed. However, for mixed-valent system with a long bridge, the electronic coupling is weak and two redox sites have very similar redox potentials. It is often difficult to determine what potential is needed to generate the mixed-valent state and the stability of the mixed-valent compound is rather low because of comproportionation (the potential splitting issue). The comproportionation constant *K*_c_ for the equation [M^n^-BL-M^n^] + [M^n+1^-BL-M^n+1^] → 2[M^n^-BL-M^n+1^] (M stands for the redox site; BL stands for the bridging ligand) is determined by *K*_c_ = 10^(Δ*E*(mV)/59)^ for a room temperature case. A small Δ*E* will lead to a high degree of proportionation of the mixed-valent compound [M^n^-BL-M^n+1^] into the homo-valent compounds [M^n^-BL-M^n^] and [M^n+1^-BL-M^n+1^].

To overcome these two issues, we present herein a “hybridized redox-asymmetry” method, instead of the common mixed-valence chemistry, for probing the electronic communication in the long-distance regime. As a proof of concept, the electronic coupling between the redox active amine site (organic) and cyclometalated ruthenium ion (inorganic) bridged by the *para*-oligophenylene wire has been examined (complexes **1**–**6** in Fig. 1). Mixed-valent compounds with either amine^23^,^24^,^25^ or cyclometalated ruthenium complexes^26^,^27^,^28^,^29^ as the redox sites have been previously investigated. The asymmetric structures of **1**–**6** and their proligands **13**–**18** will be helpful in improving the solubility of these compounds and thus making easier the synthesis, characterization, and analysis of these materials. Triarylamine and cyclometalated ruthenium have similar, but not identical, N^•+/0^ and Ru^III/II^ potentials^30^,^31^. The similar redox potentials of two species are beneficial for a strong electronic coupling between them. The nonidentical redox potentials will lead to a distinct electrochemical potential splitting, which is helpful for approaching and stabilizing the one-electron-oxidized state and thus identifying possible weak donor-to-acceptor charge transfer transition for long congeners.

## Results

In order to study the odd-electron compounds **1**^2+^–**6**^2+^, complexes **1**(PF_6_)–**6**(PF_6_) have been synthesized, by the treatment of [Ru(tpy)Cl_3_] (tpy = 2,2′:6′,2″-terpyridine) with ligands **13**–**18**, respectively, in the presence of AgOTf and followed by anion exchange using KPF_6_. The synthesis of complex **1**(PF_6_) and ligand **13** have been previously reported^30^. Ligands **14**–**18** were prepared by the Suzuki coupling between corresponding boronic acid (**7**, **8**, or **9**) and aryl bromide (**10**, **11**, or **12**). The synthesis and characterization of new compounds are given in the Experimental Section. These ligands and complexes have good solubility in CHCl_3_ and CH_2_Cl_2_ and allow the full characterization and following electrochemical and spectroscopic analysis.

**Figure 2:**
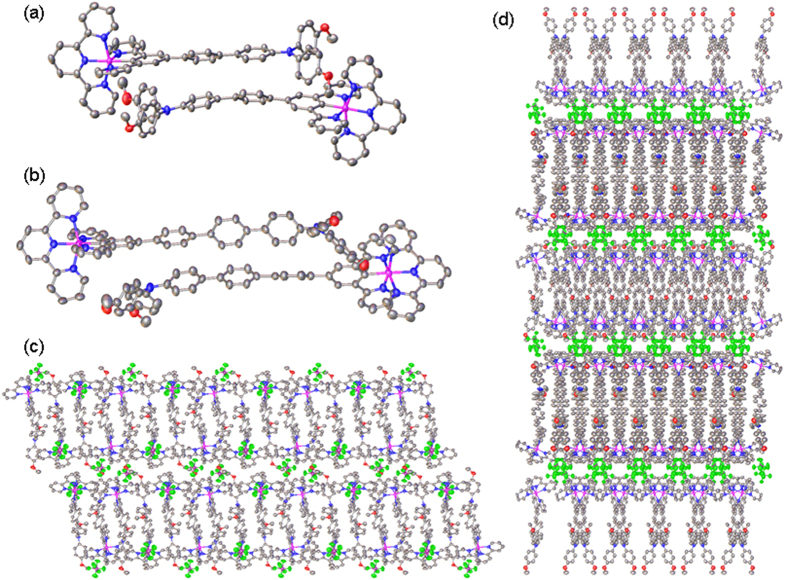
Dimeric crystal packing of (a) 3(PF_6_) and (b) 4(PF_6_) and lamellar crystal packing of (c) 3(PF_6_) (view from a axis) and (d) 4(PF_6_) (view from c axis). Anions in (**a**,**b**) and H atoms are omitted for clarity. Color code: carbon, grey; nitrogen, blue; oxygen, red; ruthenium, magenta; phosphorous, pink; fluoro, green.

The chemical structures of **3**(PF_6_) and **4**(PF_6_) are also confirmed by the single-crystal X-ray analysis (Fig. 2 and Table S1). Interestingly, both complexes show a head-to-tail anti-parallelly aligned dimeric structure. The cavity formed by two terminal *p*-anisyl groups of one molecule is used to accommodate one tpy pyridine unit of another molecule. The two molecules are held together by the van der Walls interaction between two bridging oligophenylene units. The two molecules in the dimeric structure have slightly different conformation. One molecule has essentially a linear oligophenylene unit. Another is slightly bended. The Ru-N distance of the linear molecule is 14.91 and 19.23 Å for **3**(PF_6_) and **4**(PF_6_), respectively. On the basis of the dimeric structure, lamellar crystal packing is observed in both complexes. The lamellar packing of **4**(PF_6_) is particularly interesting in that the counteranions (PF_6_^−^) form as an independent and well-separated layer (the green layer in Fig. 2d) from the anti-parallelly aligned dimeric layers. Such crystal structures are potentially useful in crystal engineering.

**Figure 3:**
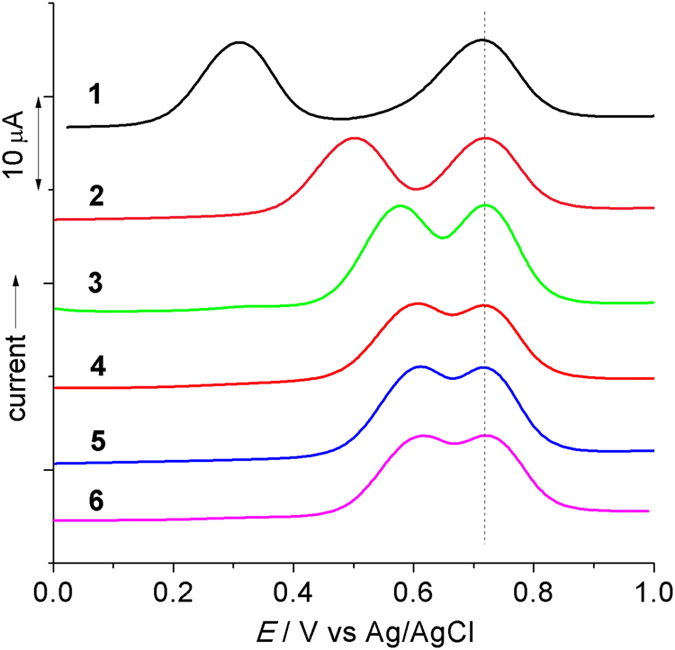
Anodic DPVs of 1(PF_6_)–6(PF_6_) in 0.1 M Bu_4_NClO_4_/CH_2_Cl_2_. *E*_1/2_ = +0.310 and +0.720 V for 1, +0.500 and +0.720 V for 2, +0.574 and +0.717 V for 3, +0.606 and +0.718 V for 4, +0.611 and +0.718 V for 5, and +0.616 and +0.721 V for 6, respectively. The potential splitting is 410, 220, 143, 112, 107, and 105 mV for **1**–**6**, respectively.

Figure 3 shows the anodic differential pulse voltammograms (DPVs) of **1**(PF_6_)–**6**(PF_6_) in CH_2_Cl_2_. Two waves are observed for all complexes in the potential region between +0.2 and +0.9 V vs Ag/AgCl. The potential of the second wave remains essentially constant around +0.72 V. The first wave shifts from +0.31 V for **1** to around +0.61 V for **4**–**6**. The potential splitting Δ*E* between two waves decreases from 410, 220, 143, to 112 mV through **1**–**4**. Compounds **5** and **6** have very similar potential splitting (around 110 mV) as **4**. The comproportionation constant *K*_c_ is 8.9 × 10^6^, 5400, and 260 for **1**^2+^–**3**^2+^, respectively, as determined by *K*_c_ = 10^ΔE/59(mV)^ for a room temperature case. The *K*_c_ value of **4**^2+^–**6**^2+^ is around 70. These data suggest that all compounds have good thermodynamic stability in the one-electron-oxidized state, irrespective of the length of the molecule. Figure S1 shows the cyclic voltammograms (CVs) of **1**–**6**. The two anodic redox couples have good chemical reversibility. One cathodic redox couple can be observed for all complexes, assigned to the reduction of the tpy ligand. Ligands **13**–**18** displays a N^•+/0^ process around the same potential at +0.70 V (Figure S2).

It should be pointed out that the potential splitting of this system is caused by many factors, including the contributions from the resonance exchange, the energy difference (Δ*G*^0^) between the reactant and product states of the electron transfer process, the electrostatic effect, the inductive effect, among others^32^,^33^. For short congeners with strong electronic coupling, the contributions from the resonance exchange play a more important role. For long congeners with weak coupling, the contribution from the redox asymmetry Δ*G*^0^ is believed to play a major role.

**Figure 4:**
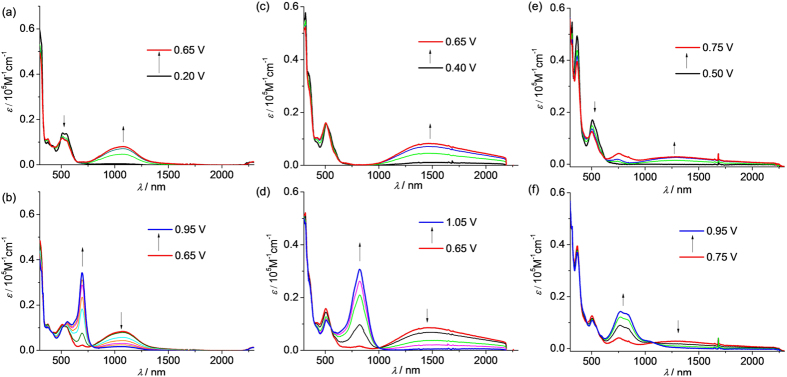
Absorption spectral changes of (a,b) 1(PF_6_), (c,d) 2(PF_6_), and (e,f) 3(PF_6_) upon (a,c,e) single- and (b,d,f) double-oxidation in 0.1 M Bu_4_NClO_4_/CH_2_Cl_2_ by stepwise electrolysis using an ITO glass electrode. The applied potentials shown in the insets are referenced vs Ag/AgCl.

**Figure 5:**
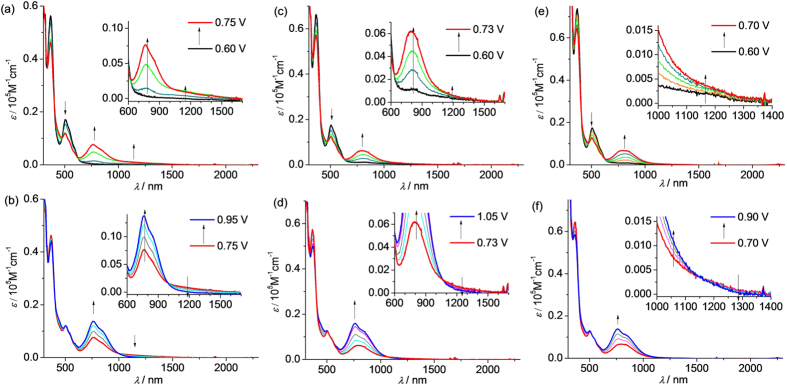
Absorption spectral changes of (a,b) 4(PF_6_), (c,d) 5(PF_6_), and (e,f) 6(PF_6_) upon (a,c,e) single- and (b,d,f) double-oxidation in 0.1 M Bu_4_NClO_4_/CH_2_Cl_2_ by stepwise electrolysis using an ITO glass electrode. The applied potentials shown in the insets are referenced vs Ag/AgCl. The insets show the enlarged spectral changes in the NIR region.

The distinct potential splitting of the two anodic redox waves of **1**(PF_6_)–**6**(PF_6_) allows the stepwise oxidative electrolysis proceed smoothly to approach the odd-electron state **1**^2+^–**6**^2+^. Figures 4 and 5 show the absorption spectral changes during the one-electron (single) and the second one-electron (double) oxidation processes of these compounds at a transparent indium-tin-oxide (ITO) glass electrode in CH_2_Cl_2_. In all cases, the donor-to-acceptor charge transfer transition in the near-infrared (NIR) region could be identified, which appeared in the single-oxidation step and decreased upon the double-oxidation. For the short congeners **1**(PF_6_)–**3**(PF_6_) (Fig. 4), these changes are very clear and the intense charge transfer transitions are well-separated from the newly formed absorptions in the region of 600∼1000 nm. These latter absorption bands are mainly assigned to the N^•+^-localized transitions^23^,^24^,^25^. This band is also observed when the amine ligand, e.g. compound **14**, was electrolyzed (Figure S3).

For the long congeners **4**(PF_6_)–**6**(PF_6_) (Fig. 5), the charge transfer transitions are less obvious. However, the enlarged plots of the NIR spectral changes clearly show that lower-energy edge absorptions around 1000 nm increased upon single-oxidation and decreased upon double-oxidation. This strongly supports the presence of the donor-to-acceptor charge transfer transitions in the odd-electron compounds **4**^2+^–**6**^2+^, which overlap severely with the absorption bands between 600 and 1000 nm. For the long congeners **4**^2+^–**6**^2+^, the absorption bands between 600 and 1000 nm contain both the N^•+^-localized transitions and the bridge-to-aminium charge transfer transitions. Similar absorptions can be observed when ligand **16** was stepwisely oxidized by electrolysis (Figure S4), and the assignments are supported by TDDFT results of **16**^+^ (Figure S5).

**Figure 6:**
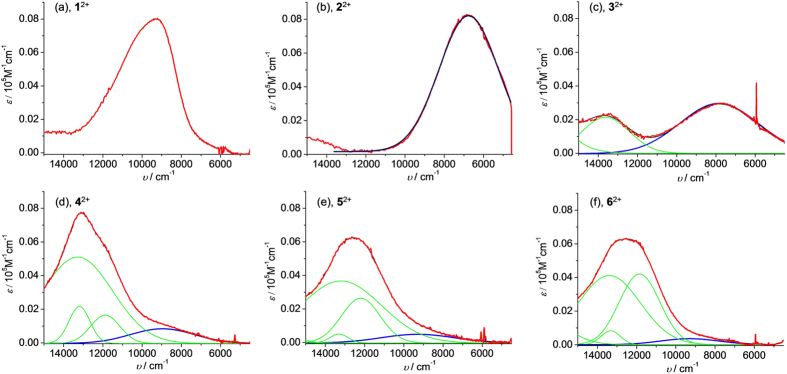
NIR absorptions of (a-f) 1^2+^ through 6^2+^ in CH_2_Cl_2_ recorded during the spectroelectrochemical measurements. The donor-to-acceptor charge transfer transitions of **2**^2+^– **6**^2+^ are highlighted in the blue color after fitting with Gaussian functions. The red curves are the experimental data. The black curves are the sum of the deconvoluted curves.

The donor-to-acceptor charge transfer transition of **1**^2+^–**6**^2+^ is not intervalence charge transfer in nature. However, the Hush analysis, originally developed for the treatment of mixed-valent system^34^, can be used to estimate the donor-acceptor coupling of these complexes. Similar analysis has been performed by Creutz, Newton, Sutin, Crutchley, and others^35^,^36^,^37^,^38^, provided that the transition dipole lies along the donor-acceptor axis and donor-acceptor overlap can be ignored. The NIR absorptions of **1**^2+^–**6**^2+^ are plotted against wavenumbers (Fig. 6). The charge transfer transition of **1**^2+^ is asymmetric. The bandwidth of the high-energy side is twice the width of the low-energy side. The charge-transfer transitions of **2**^2+^ and **3**^**2**+^ are Gaussian-symmetric and well-separated from the visible absorptions (Fig. 6b,c).

The potential donor-to-acceptor charge transfer transitions of **4**^2+^–**6**^2+^ are overlapped with low-energy edge of the N^•+^-bands and the bridge-to-aminium charge transfer transitions. The NIR absorptions of these compounds are fitted to a few Gaussian functions and the deconvoluted donor-to-acceptor charge transfer transitions are shown in the blue color in Fig. 6b–f (for **2**^2+^ through **6**^2+^, respectively).

The electronic coupling parameter *V*_ab_ was calculated to be 880, 450, 180, 120, and 80 cm^−1^ for **2**^2+^–**6**^2+^, respectively (Table 1), using a similar Hush formula *V*_ab_ = 0.0206 × (ε_max_*υ*_max_Δν_1/2_)^1/2^/(*R*_ab_)^34^, where the electron transfer distance *R*_ab_ was taken to be the DFT calculated Ru-N geometrical distance. For complex **1**^2+^, the charge transfer transition is asymmetric. The *V*_ab_ values was calculated to be 1530 cm^−1^ by *V*_ab_ = (μ_ge_*υ*_max_)/e*R*_ab_, where *μ*_ge_ is the transition dipole moment of the charge transfer band and *e* is the elementary charge. This equation is applicable to charge transfer band of any shape and *μ*_ge_ can be calculated from the integrated absorbance of the charge transfer band^23^.

For a series of related donor-acceptor compounds with a tunneling-dominated mechanism, the distance dependence of resonance exchange is characterized by an exponential decay, *V*_ab_ = *V*^0^_ab_exp(−*βR*_ab_)^16^,^17^,^18^,^19^,^20^, where *V*_ab_ and *V*^0^_ab_ are the electronic coupling parameter at distance *R*_ab_ and van der Waals contact, respectively, and *β* is the decay factor. In some literatures^23^,^39^, *V*_ab_ = *V*^0^_ab_exp(−*β*_2_*R*_ab_/2) is applied with the factor of two in the denominator of the exponential term, considering that the rate constant for electron transfer is proportional to *V*_ab_^2^ when *V*_ab_ is small.

**Figure 7:**
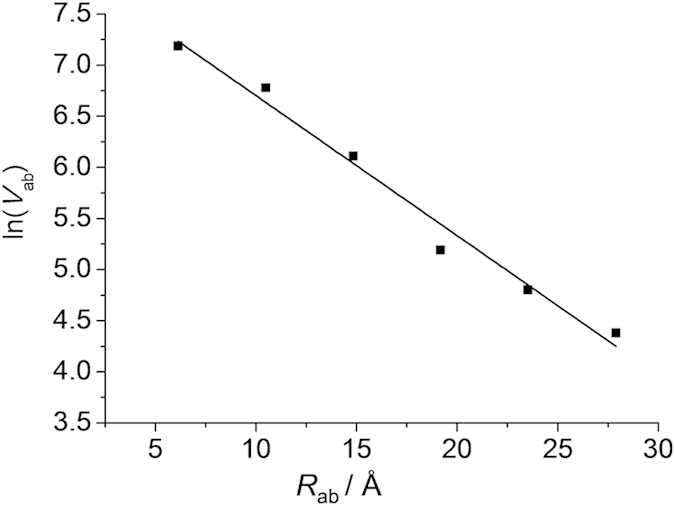
Distance dependence plot of ln(V_ab_) as a function of *R*_ab_ (Å) from data in Table 1. The data was fitted to a linear equation with a slope of −0.137 Å^−1^ and adjusted R^2^ of 0.978.

Figure 7 shows the distance dependence plot of ln(*V*_ab_) versus *R*_ab_ from data in Table 1, where the six data can be well fitted to a negative linear equation with a decay slope of −0.137 Å^−1^ (*β* = 0.137*; β*_2_ = 0.274) and a *V*^0^_ab_ value of 3204 cm^−1^. This means that the long congeners **5**^2+^ and **6**^2+^ are still in the regime of tunneling superexchange. The linear correlation with the inclusion of **1**^2+^ suggests that the Hush analysis is still applicable to **1**^2+^, althouth it may have a strong donor-acceptor orbital overlap. The decay slope of the **1**^2+^–**6**^2+^ series is comparable to that of the oligophenylene-bridged cyclometalated diruthenium series (*β* = 0.118 Å^−1^)^16^ and bis-triarylamine series (*β*_2_ = 0.32 Å^−1^)^23^.

**Figure 8:**
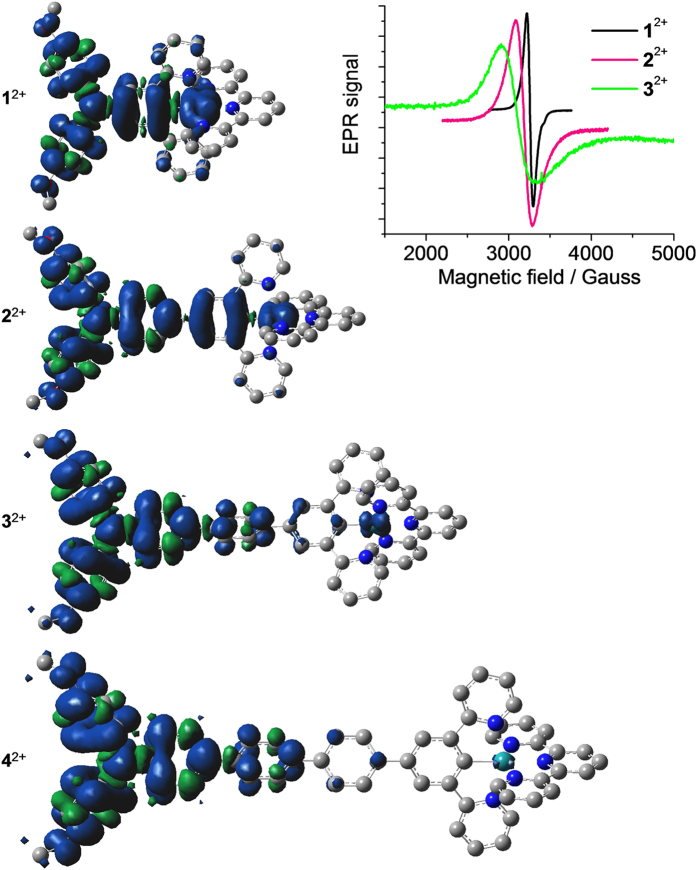
DFT calculated spin density plots of 1^2+^–4^2+^ and EPR signals of 1^2+^–3^2+^. DFT methods: UB3LYP/LANL2DZ/6-31-G*/CPCM. Samples **1**^2+^–**3**^2+^ (for EPR measurement) were obtained by oxidation of **1**(PF_6_)–**3**(PF_6_) (0.01 M) with 0.5 equiv cerium ammonium nitrate in CH_3_CN.

DFT calculations show that the free spins of **1**^2+^ and **2**^2+^ are delocalized across the amine-bridge-ruthenium array (Fig. 8). In comparison, the spin contribution of the ruthenium ion of **3**^2+^ decreases significantly. The Mulliken contribution of ruthenium is 0.220, 0.170, and 0.058 for **1**^2+^–**3**^2+^, respectively (Figure S6). From complex **4**^2+^ through **6**^2+^, the spin becomes localized on the triarylamine segment (Fig. 8 and S7). Complexes **1**^2+^–**3**^2+^ all show a single-line isotropic EPR signal with the *g* value of 2.086, 2.131, and 2.175, respectively (shown in the upper-right corner of Fig. 8). The EPR signal becomes increasingly broad through **1**^2+^ to **3**^2+^, which may be caused by a longer spin delocalization distance of **3**^2+^ with respect to those of **1**^2+^ and **2**^2+^. Complex **4**^2+^ displays a similar broad EPR signal as **3**^2+^ (Figure S8). The broad EPR signals could also be caused by the intermolecular electron exchange between diamagnetic and radical species. Nevertheless, these results suggest that the free spin of **1**^2+^–**3**^2+^ is more biased toward the amine unit, while the free spin of **4**^2+^–**6**^2+^ is essentially localized on the amine unit. The donor-to-acceptor charge transfer transition of these compounds can be assigned to the ruthenium (2+) to aminium (N^•+^) charge transfer.

The involved electron transfer is believed to occur via a hole-transfer superexchange mechanism^40^,^41^, as has been proposed in symmetric mixed valent compounds with either triarylamine^23^,^24^,^25^ or cyclometalated ruthenium^26^,^27^,^28^,^29^ as the charge-bearing sites. This assertion is also supported by DFT calculations. Taking complex **4**^2+^ as an example, the HOMO and HOMO-1 orbital is localized on the triarylamine or cyclometalated ruthenium segment, respectively (Figure S9). The highest bridge-dominated occupied orbital is HOMO-4, which is around 1.2 eV lower relative to HOMO. In comparison, the energy gap between the lowest bridge-dominated unoccupied orbital (LUMO+6) and the donor or acceptor orbital (HOMO and HOMO-1) is much larger (>3.0 eV), which raises a high barrier to the electron-transfer superexchange.

In conclusion, we have demonstrated the long-distance electron transfer in a series of cyclometalated ruthenium-amine odd-electron compounds, which decreases exponentially with a decay slope of −0.137 Å^−1^ through the para-oligophenylene wire. Donor-acceptor charge transfer transition has been observed for a compound with a separation of 27.85 Å between two redox sites. This is one of the charge-transfer transitions involving electron transfer over the longest distance to date. By using the hybrid of the redox-asymmetric inorganic and organic redox species, the solubility and potential-splitting issues of long symmetric mixed valent compounds are suppressed. The asymmetric structures of the bridging ligands and corresponding complexes improve the solubility of long congeners. The similar but nonidentical N^•+/0^ and Ru^III/II^ redox potentials leads to the presence of weak electronic coupling and distinct electrochemical potential splitting even at a even large N-Ru separation. Such a “hybridized redox-asymmetry” method would be very useful for the design and investigation of new donor-acceptor systems with a long-distance bridge.

## Methods

### Spectroscopic Measurements

Spectroelectrochemical measurements were performed in a thin layer cell (optical length = 0.2 cm), in which an ITO glass electrode (<10Ω/square) working electrode was set in CH_2_Cl_2_ containing the compound to be studied (concentration around 5 × 10^−5^ M) and 0.1 M Bu_4_NClO_4_ as the supporting electrolyte. A platinum wire and Ag/AgCl in saturated aqueous NaCl solution was used as the counter electrode and reference electrode, respectively. The cell was put into a PE Lambda 750 UV/vis/NIR spectrophotometer to monitor spectral changes during electrolysis.

### Electrochemical Measurements

All electrochemical measurements were taken using a CHI 660D potentiostat with one-compartment electrochemical cell under an atmosphere of nitrogen. All measurements were carried out in 0.1 M Bu_4_NClO_4_ in CH_2_Cl_2_. The working electrode was a glassy carbon with a diameter of 3 mm. The electrode was polished prior to use with 0.05 μm alumina and rinsed thoroughly with water and acetone. A large area platinum wire coil was used as the counter electrode. All potentials are referenced to a Ag/AgCl electrode in saturated aqueous NaCl. The potentials have not been compensated for the liquid junction. The potential versus ferrocene^+/0^ can be subtracted by 0.45 V.

### X-ray Crystallography

The X-ray diffraction data were collected using a Rigaku Saturn 724 diffractometer on a rotating anode (Mo-K radiation, 0.71073 Å) at 173 K. The structure was solved by the direct method using SHELXS-97 and refined with Olex2. CCDC 1031690 and 1031691 contain the supplementary crystallographic data for this paper. These data can be obtained for free of charge from the Cambridge Crystallographic Data Centre via www.ccdc.cam.ac.uk/data_request/cif. The relatively large R1 and wR2 values of **4**(PF_6_) are possibly caused by the poor quality of the single crystal.

### Computational Methods

DFT calculations are carried out using the B3LYP exchange correlation functional and implemented in the *Gaussian* 09 package. The electronic structures were optimized using a general basis set with the Los Alamos effective core potential LanL2DZ basis set for Ru and 6-31G* for other atoms. The solvation effects in dichloromethane solutions are taken into account for all calculations with the conductor-like polarizable continuum model (CPCM). No symmetry constraints were used in the optimization (nosymm keyword was used). Frequency calculations have been performed with the same level of theory to ensure the optimized geometries to be local minima. All orbitals have been computed at an isovalue of 0.02 e/bohr^3^.

### EPR Measurements

EPR measurements were performed on a Bruker ELEXSYS E500-10/12 spectrometer at 77 K in frozen CH_3_CN. The spectrometer frequency is 9.7 × 10^9^ Hz. Samples **1**^2+^–**4**^2+^ were obtained by oxidation of **1**(PF_6_)–**4**(PF_6_) with 0.5 equiv cerium ammonium nitrate in CH_3_CN.

## Additional Information

**How to cite this article**: Shen, J.-J. and Zhong, Y.-W. Long-Range Ruthenium-Amine Electronic Communication through the *para*-Oligophenylene Wire. *Sci. Rep.* **5**, 13835; doi: 10.1038/srep13835 (2015).

## Supplementary Material

Supporting information

## Figures and Tables

**Figure 1:**
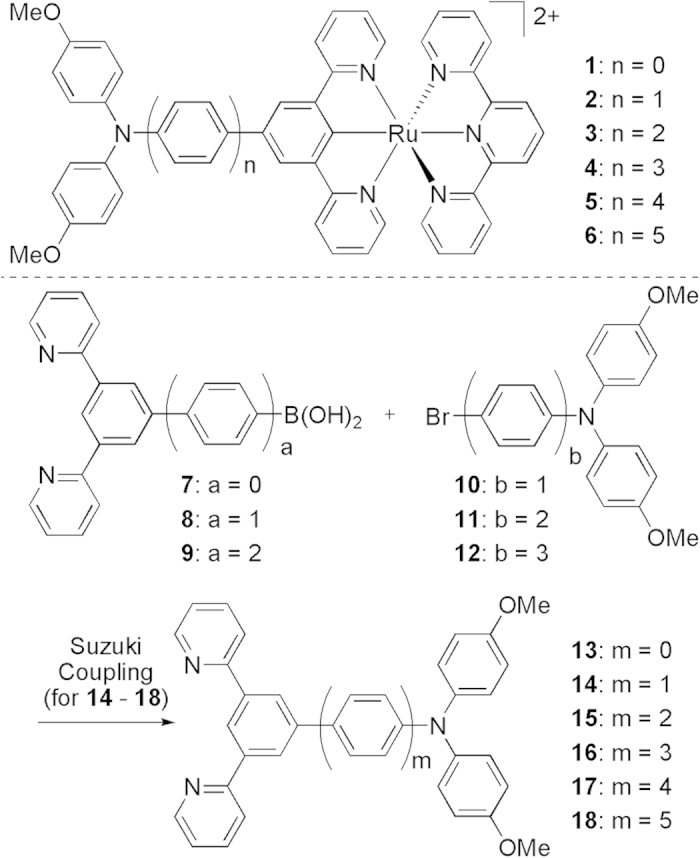
Chemical structures of complexes 1^2+^–6^2+^ and the synthetic route to ligands 14–18.

**Table 1:** Parameters for donor-to-acceptor charge transfer transitions^a^.

	*υ*_max_(cm^−1^)	*ε*_max_(M^−1^cm^−1^)	Δ*υ*_1/2_(cm^−1^)^b^	*R*_ab_^c^(Å)	*V*_ab_^d^(cm^−1^)
**1**^2+^	9250	8000	3400	6.13	1530
**2**^2+^	6780	8200	3620	10.48	880
**3**^2+^	7810	2900	4730	14.84	450
**4**^2+^	8960	840	3730	19.18	180
**5**^2+^	9260	490	4310	23.52	120
**6**^2+^	9300	360	3580	27.85	80

^a^Based on the spectroelectrochemical results in CH_2_Cl_2_.

^b^The experimentally observed width at half-height.

^c^Estimated by the DFT-optimized Ru-N geometrical distance.

^d^*V*_ab_ values calculated by (μ_ge_*υ*_max_)/eR for **1**^2+^ and 0.0206 × (ε_max_*υ*_max_Δν_1/2_)^1/2^/(*R*_ab_) for **2**^2+^–**6**^2+^.
