# Supplementary material for: Long-Range Ruthenium-Amine Electronic Communication through the para-Oligophenylene Wire
Source: Sci Rep. 2015 Sep 7;5:13835. doi: 10.1038/srep13835 (PMC4561373; doi:10.1038/srep13835)

Supporting Information for:

Long-Range Ruthenium-Amine Electronic Communication through the *para*-Oligophenylene Wire

Jun-Jian Shen and Yu-Wu Zhong*

Beijing National Laboratory for Molecular Science, CAS Key Laboratory of Photochemistry, Institute of Chemistry, Chinese Academy of Sciences, Beijing 100190, People's Republic of China;

*Email: zhongyuwu@iccas.ac.cn

Synthesis. All reagents were purchased from commercial suppliers and used without further purification unless otherwise specified. All the reactions dealing with air- and moisture-sensitive compounds were carried out in a dry reaction vessel under positive pressure of nitrogen. Proton nuclear magnetic resonance (^{1}H NMR) and carbon nuclear magnetic resonance (^{13}C NMR) spectra were measured on BRUKER AVANCE 300 and BRUKER DMX 400 spectrometers. Chemical shifts for hydrogen are reported in parts per million (ppm, δ scale) downfield from tetramethylsilane and are referenced to the residual protons in the NMR solvent ($CDCl_3$: δ 7.26, CD_3OCD_3, δ 2.05, CD_3CN, 1.92). ^{13}C NMR spectra were recorded at 100 MHz or 75 MHz. Chemical shifts for carbons are reported in parts per million (ppm, δ scale) downfield from tetramethylsilane and are referenced to the carbon resonance of the solvent ($CDCl_3$: δ 77.0, CD_2Cl_2: δ 53.84). The data are presented as follows: chemical shift, multiplicity (s = singlet, d = doublet, t = triplet, m = multiplet and/or multiple resonances, br = broad), coupling constant in Hertz (Hz), and integration. MS data were obtained with a Bruker Daltonics Inc. ApexII FT-ICR or Autoflex III MALDI-TOF mass spectrometer. The matrix for MALDI-TOF measurement is α-cyano-4-hydroxycinnamic acid. Microanalysis was carried out using Flash EA 1112 or Carlo Erba 1106 analyzer at the Institute of Chemistry, Chinese Academy of Sciences. Complex **1**(PF_6) and ligand **13**[1] and bromides **10** and **11** were prepared according to the known procedures.[2]

Synthesis of Boronic Acid 7. To a solution of 3,5-di(pyrid-2-yl)-bromobenzene[1] (311 mg, 1.0 mmol) in 10 mL dry tetrahydrofuran was added dropwisely *n*-BuLi/*n*-hexane (0.44 mL, 1.1 mmol, 2.5 M) at -78°C. The resulting solution was stirred at this temperature for 0.5 h. Trimethyl borate (156 mg, 1.5 mmol) was then added and the system was stirred for another 0.5 h. The reaction was quenched with dilute HCl (20%, 2 mL), and the mixture was concentrated by rotary evaporation. The crude product was dissolved in 5 mL methanol, followed by the addition of proper amount of Et_2O. The resulting precipitate was collected by filtering and washing with Et_2O to give 210 mg of 7 as a white solid in 76% yield. This sample was used directly in the next step without further purification. ^{1}H NMR (300 MHz, D_2O): δ 7.95 (t, J = 6.9 Hz, 2H), 8.26 (d, J = 8.1 Hz, 2H), 8.31 (s, 1H), 8.34 (s, 2H), 8.57 (t, J = 8.1 Hz, 2H), 8.72 (d, J = 5.7 Hz, 2H). ^{13}C NMR (75 MHz, CD_3OD): δ 126.03, 126.49, 129.80, 131.73, 136.50, 142.13, 147.20, 151.61.

Synthesis of 1,3-Di(pyrid-2-yl)-5-(*para*-bromophenyl)benzene. To a suspension of 4-bromo-1-iodobenzene (142 mg, 0.5 mmol), $Pd(PPh_3)_4$ (29 mg, 0.025 mmol), and

[1] (a) C.-J. Yao, R.-H. Zheng, Q. Shi, Y.-W. Zhong and J. Yao, *Chem. Commun.*, 2012, **48**, 5680; (b) C.-J. Yao, H.-J. Nie, W.-W. Yang, J.-Y. Shao, J. Yao and Y.-W. Zhong, *Chem. Eur. J.*, 2014, **20**, 17466.
[2] Y. J. Chang and T. J. Chow, *Tetrahedron*, 2009, **65**, 9626.

K_2CO_3 (345 mg, 2.5 mmol) in a mixed solvent of degassed THF/H_2O (4.5 mL/0.5 mL) was added boronic acid **7** (166 mg, 0.6 mmol). The mixture was stirred at 90 °C for 12 h. The solvent was evaporated under reduced procedure. The crude product was purified by column chromatography on silica gel (eluent: CH_2Cl_2/EtOAc, 15/1) to give 210 mg of 1,3-di(pyrid-2-yl)-5-(*para*-bromophenyl)benzene as a white solid in 55% yield. ^{1}H NMR (400 MHz, CD_3CN): δ 7.36 (dd, J = 7.4, 5.0 Hz, 2H), 7.68 (d, J = 8.8 Hz, 2H), 7.74 (d, J = 8.8 Hz, 2H), 7.89 (td, J = 8.0, 1.6 Hz, 2H), 8.03 (d, J = 8.0 Hz, 2H), 8.33 (d, J = 1.2 Hz, 2H), 8.72 (d, J = 4.8 Hz, 2H), 8.78 (s, 1H). ^{13}C NMR (100 MHz, $CDCl_3$): δ 120.88, 121.86, 122.53, 124.77, 126.15, 129.01, 131.88, 136.88, 139.82, 140.61, 141.14, 149.77, 156.96. HRMS (EI) calcd for $C_{22}H_{15}N_2Br$ ($[M]^+$): 386.0419. Found 386.0419.

Synthesis of Boronic Acid 8. This compound (white solid) was prepared from 1,3-di(pyrid-2-yl)-5-(*para*-bromophenyl)benzene in 75% yield, using the similar procedure for the synthesis of boronic acid **7**. ^{1}H NMR (400 MHz, CD_3OD): δ 7.88 (br, 2H), 7.93 (d, J = 7.6 Hz, 2H), 8.13 (t, J = 6.6 Hz, 2H), 8.58 (m, 3H), 8.64 (d, J = 8.0 Hz, 2H), 8.76 (t, J = 8.0 Hz, 2H), 8.97 (d, J = 5.6 Hz, 2H). ^{13}C NMR (100 MHz, CD_3OD): δ 126.17, 126.21, 126.59, 127.15, 129.67, 132.94, 134.43, 139.31, 142.16, 143.82, 147.11, 151.05.

Synthesis of 1,3-Di(pyrid-2-yl)-5-(4″-bromobiphen-4′-yl)benzene. This compound (white solid) was prepared from boronic acid **7** and 4,4′-dibromobiphenyl in 72% yield, using the similar procedure for the synthesis of 1,3-di(pyrid-2-yl)-5-(*para*-bromophenyl)benzene. ^{1}H NMR (300 MHz, $CDCl_3$): δ 7.30 (m, 2H), 7.53 (d, J = 8.7 Hz, 2H), 7.60 (d, J = 8.4 Hz, 2H), 7.85 (m, 4H), 7.92 (d, J = 7.8 Hz, 2H), 8.35 (d, J = 1.8 Hz, 2H), 8.60 (t, J = 1.8 Hz, 1H), 8.76 (d, J = 4.8 Hz, 2H). ^{13}C NMR (100 MHz, $CDCl_3$): δ 120.86, 121.65, 122.45, 124.67, 126.25, 127.27, 127.89, 128.65, 131.94, 136.83, 139.11, 139.61, 140.22, 140.56, 141.64, 149.76, 157.13. HRMS (MALDI-TOF) calcd for $C_{28}H_{20}BrN_2$ ($[M + H]^+$): 463.0810. Found: 463.0804.

Synthesis of Boronic Acid 9. This compound was prepared from 1,3-di(pyrid-2-yl)-5-(4″-bromobiphen-4′-yl)benzene in 70% yield, using the similar procedure for the synthesis of boronic acid **7**. ^{1}H NMR (400 MHz, CD_3OD): δ 7.70 (d, J = 8.0 Hz, 2H), 7.79 (br, 2H), 7.87 (d, J = 8.0 Hz, 2H), 8.04 (d, J = 8.0 Hz, 2H), 8.15 (t, J = 6.8 Hz, 2H), 8.62 (m, 3H), 8.66 (d, J = 8.0 Hz, 2H), 8.77 (td, J = 7.6, 0.8 Hz, 2H), 8.98 (d, J = 6.0 Hz, 2H). ^{13}C NMR (100 MHz, CD_3OD): δ 125.59, 126.13, 126.52, 126.93, 127.33, 127.64, 129.40, 132.82, 134.16, 136.82, 141.01, 141.16, 142.13, 143.23, 147.06, 150.89.

Synthesis of Bromide 12. To a solution of 4,4''-dibromo-1,1':4',1''-terphenyl (776 mg, 2.0 mmol) in 60 mL dry toluene were added $Pd_2(dba)_3$ (37 mg, 0.1 mmol), dppf (55 mg, 0.1 mmol), NaOBu^t (576 mg, 6.0 mmol), and bis(4-methoxyphenyl)amine (504 mg, 2.2 mmol). The solution was bubbled for 10 minutes with nitrogen and then heated at 110 ºC for 12 h. After cooling to room temperature, the solvent was evaporated and the residue was purified by column chromatography on silica gel (eluent: petroleum ether/CH_2Cl_2, 5/4) to give 190 mg of bromide **12** in 20% yield as a pale yellow solid. ^{1}H NMR (400 MHz, Acetone-d$_6$): δ 3.80 (s, 6H), 6.93 (d, J = 8.8 Hz, 6H), 7.10 (d, J = 8.8 Hz, 4H), 7.56 (d, J = 8.8 Hz, 2H), 7.60-7.80 (m, 8H). ^{13}C NMR (100 MHz, CD_2Cl_2): δ 56.71, 122.57, 128.13, 128.39, 128.53, 129.78, 130.08, 133.13, 140.90. HRMS (MALDI-TOF) calcd for $C_{32}H_{26}NO_2Br$ ($[M]^+$): 535.1147. Found: 535.1141.

Synthesis of Ligand 14. This compound was synthesized according to a modified known procedure. To a suspension of bromide **10** (166 mg, 0.5 mmol), $Pd(PPh_3)_4$ (29 mg, 0.025 mmol), K_2CO_3(345 mg, 2.5 mmol) in degassed THF/H_2O (4.5 mL/0.5 mL) was added boronic acid **7** (166 mg, 0.6 mmol). The mixture was stirred for 12 h at 90 °C. The solvent was evaporated in vacuum and the crude product was purified by column chromatography on silica gel (eluent: CH_2Cl_2/EtOAc, 15/1) to give 139 mg ligand **14** in 52% yield as a pale yellow solid. ^{1}H NMR (300 MHz, $CDCl_3$): δ 3.81 (s, 6H), 6.85 (d, J = 8.8 Hz, 2H), 7.03 (d, J = 8.4 Hz, 2H), 7.10 (d, J = 8.8 Hz, 4H), 7.20-7.30 (m, 2H), 7.58 (d, J = 8.4 Hz, 2H), 7.79 (t, J = 7.2 Hz, 2H), 8.25 (s, 2H), 7.89 (d, J = 8.4 Hz, 2H), 8.53 (s, 1H), 8.73 (d, J = 3.6 Hz, 2H). ^{13}C NMR (100 MHz, $CDCl_3$): δ 55.52, 114.71, 120.86, 120.89, 122.33, 123.79, 125.83, 126.54, 127.89, 132.88, 136.81, 140.34, 140.93, 142.06, 148.36, 149.68, 155.85, 157.34.

Synthesis of Ligand 15. This compound was prepared from boronic acid **7** and bromide **11** in 51% yield as a pale yellow solid, using the similar procedure for the synthesis of ligand **14**. ^{1}H NMR (300 MHz, $CDCl_3$): δ 3.81(s, 6H), 6.84 (d, J = 3.2 Hz, 2H), 6.87 (d, J = 3.2 Hz, 2H), 7.02 (d, J = 8.4 Hz, 2H), 7.09 (d, J = 3.6 Hz, 2H), 7.12 (d, J = 3.2 Hz, 2H), 7.20-7.30 (m, 2H), 7.49 (d, J = 8.8 Hz, 2H), 7.66 (d, J = 8.0 Hz, 2H), 7.75-7.85 (m, 4H), 7.91 (d, J = 8.0 Hz, 2H), 8.34 (d, J = 1.2 Hz, 2H), 8.59 (s, 1H), 8.75 (d, J = 4.4 Hz, 2H). ^{13}C NMR (100 MHz, $CDCl_3$): δ 55.52, 114.73, 120.74, 120.93, 122.42, 124.44, 126.21, 126.65, 126.77, 127.46, 127.70, 132.43, 136.86, 138.92, 140.03, 140.46, 140.87, 141.95, 148.26, 149.73, 155.91, 157.22. HRMS (MALDI-TOF) calcd for $C_{42}H_{33}N_3O_2$ ($[M]^+$): 611.2573. Found: 611.2567.

Synthesis of Ligand 16. This compound was prepared from boronic acid **7** and bromide **12** in 44% yield as a pale yellow solid, using the similar procedure for the synthesis of ligand **14**. ^{1}H NMR (400 MHz, Acetone-d$_6$): δ 3.81 (s, 6H), 6.90-7.00 (m,

6H), 7.10 (m, 4H), 7.40 (dd, *J* = 7.2, 5.2 Hz, 2H), 7.59 (d, *J* = 8.4 Hz, 2H), 7.75 (d, *J* = 8.4 Hz, 2H), 7.82 (d, *J* = 8.0 Hz, 2H), 7.92-8.00 (m, 6H), 8.19 (d, *J* = 8.0 Hz, 2H), 8.54 (d, *J* = 1.6 Hz, 2H), 8.75 (d, *J* = 4.4 Hz, 2H), 8.91 (s, 1H). ^{13}C NMR (100 MHz, CD_2Cl_2): δ 55.44, 114.67, 120.41, 120.61, 122.50, 124.41, 125.95, 126.68, 126.75, 127.21, 127.24, 127.26, 127.68, 130.02, 136.83, 138.45, 139.66, 139.78, 139.83, 140.44, 140.72, 141.54, 148.41, 149.73, 156.14, 156.81. HRMS (MALDI-TOF) calcd for $C_{48}H_{37}N_3O_2$ ($[M]^+$): 687.2886. Found: 687.2880.

Synthesis of Ligand 17. This compound was prepared from boronic acid **8** and bromide **12** in 61% yield as a pale yellow solid, using the similar procedure for the synthesis of ligand **14**. ^{1}H NMR (300 MHz, Acetone-d_6): δ 3.81 (s, 6H), 6.80-7.05 (m, 6H), 7.11 (m, 4H), 7.40 (dd, *J* = 7.5, 5.1 Hz, 2H), 7.60 (d, *J* = 8.7 Hz, 2H), 7.70-8.10 (m, 14H), 8.20 (d, *J* = 7.8 Hz, 2H), 8.56 (d, *J* = 1.2 Hz, 2H), 8.76 (d, *J* = 5.1 Hz, 2H), 8.92 (s, 1H). ^{13}C NMR (100 MHz, CD_2Cl_2): δ 55.44, 114.67, 120.40, 120.61, 122.51, 124.45, 125.97, 126.67, 126.75, 127.19, 127.22, 127.25, 127.30, 127.34, 127.72, 132.01, 136.84, 138.44, 139.27, 139.64, 139.72, 139.75, 139.81, 140.45, 140.72, 141.51, 148.41, 149.74, 156.14, 156.80. HRMS (MALDI-TOF) calcd for $C_{54}H_{41}N_3O_2$ ($[M]^+$): 763.3199. Found: 763.3193.

Synthesis of Ligand 18. This compound was prepared from boronic acid **9** and bromide **12** in 44% yield as a pale yellow solid, using the similar procedure for the synthesis of ligand **14**. ^{1}H NMR (400 MHz, $CDCl_3$): δ 3.81(s, 6H), 6.86 (d, *J* = 9.2 Hz, 4H), 7.01 (d, *J* = 8.8 Hz, 2H), 7.11 (d, *J* = 9.2Hz, 4H), 7.29 (m, 2H), 7.48 (d, *J* = 8.8 Hz, 2H), 7.66 (d, *J* = 8.4 Hz, 2H), 7.71 (d, *J* = 8.4 Hz, 2H), 7.75-7.85 (m, 12H), 7.89 (d, *J* = 8.0 Hz, 2H), 7.93 (d, *J* = 8.0 Hz, 2H), 8.38 (d, *J* = 5.6 Hz, 2H), 8.62 (t, *J* = 1.2 Hz, 1H), 8.77 (d, *J* = 4.0 Hz, 2H). ^{13}C NMR (100 MHz, $CDCl_3$): δ 55.52, 114.77, 120.71, 120.89, 122.43, 124.62, 126.28, 126.68, 126.83, 127.07, 127.29, 127.34, 127.36, 127.42, 127.47, 127.85, 128.85, 132.40, 136.83, 138.65, 139.45, 139.62, 139.71, 139.79, 139.83, 139.88, 139.92, 140.54, 140.89, 141.82, 148.28, 149.76, 155.97, 157.21. HRMS (MALDI-TOF) calcd for $C_{60}H_{45}N_3O_2$ ($[M]^+$): 839.3512. Found: 839.3506.

Synthesis of Complex 2(PF_6). To 30 mL of dry acetone were added [Ru(tpy)Cl_3] (0.1 mmol, 44 mg) and AgOTf (0.3 mmol, 78 mg). The mixture was refluxed under a N_2 atmosphere for 3 h. The mixture was filtered to afford a purple black solution, and the filtrate was concentrated to dryness. To the residue were added ligand **14** (0.1 mmol, 61.2 mg), 3 mL of DMF, and 3 mL of *t*-BuOH. The resulting mixture was refluxed under microwave heating for 30 min at a power of 200 W and then another 30 min at a power of 375 W. After cooling to room temperature, an excess of aqueous KPF_6 were added. The resulting precipitate was collected by filtering and washing

with water and Et_2O. The crude solid was purified through flash column chromatography on silica gel, followed by anion exchange using KPF_6, to give 65 mg of complex **2**(PF_6) in 64% yield as a red solid. ^{1}H NMR (400 MHz, Acetone-d$_6$): δ 3.84 (s, 6H), 6.76 (t, J = 6.2 Hz, 2H), 6.98 (d, J = 8.8 Hz, 4H), 7.10-7.20 (m, 8H), 7.19 (d, J = 5.6 Hz, 2H), 7.28 (d, J = 5.2 Hz, 2H), 7.71 (td, J= 7.6, 1.2 Hz, 2H), 7.83 (td, J = 7.6, 1.2 Hz, 2H), 7.89 (d, J = 8.8 Hz, 2H), 8.40 (t, J = 8.0 Hz, 2H), 8.45 (d, J = 8.0 Hz, 2H), 8.65 (s, 2H), 8.69 (d, J = 8.0 Hz, 2H), 9.03 (d, J = 8.0 Hz, 2H). MALDI-MS (m/z): 869.1 for $[M - PF_6]^+$. Anal. Calcd for: $C_{51}H_{39}F_6N_6O_2PRu \cdot H_2O$: C, 59.36; H, 4.00; N, 8.14. Found: C, 59.47; H, 3.81; N, 8.24.

Synthesis of Complex 3(PF_6). This complex was prepared from [Ru(tpy)Cl_3] and ligand **15** as a red solid in 68% yield, using the similar procedure for the synthesis of complex **2**(PF_6). ^{1}H NMR (400 MHz, Acetone-d$_6$): δ 3.83 (s, 6H), 6.95 (t, J = 2.8 Hz, 2H), 6.90-7.10 (m, 6H), 7.10-7.20 (m, 6H), 7.21 (d, J = 5.2 Hz, 2H), 7.31 (d, J = 5.2 Hz, 2H), 7.66 (m, 2H), 7.73 (td, J = 7.2, 1.2 Hz, 2H), 7.80-7.90 (m, 4H), 8.14 (d, J = 8.0 Hz, 2H), 8.41 (t, J = 8.0 Hz, 1H), 8.50 (d, J = 8.0 Hz, 2H), 8.70 (d, J = 8.0 Hz, 2H), 8.77 (s, 1H), 9.04 (d, J = 8.0 Hz, 2H). MALDI-MS (m/z): 945.1 for $[M - PF_6]^+$. Anal. Calcd for: $C_{57}H_{43}F_6N_6O_2PRu \cdot H_2O$: C, 61.79; H, 4.09; N, 7.58. Found: C, 61.90; H, 4.28; N, 7.72.

Synthesis of Complex 4(PF_6). This complex was prepared from [Ru(tpy)Cl_3] and ligand **16** as a red solid in 67% yield, using the similar procedure for the synthesis of complex **2**(PF_6). ^{1}H NMR (300 MHz, Acetone-d$_6$): δ 3.83 (s, 6H), 6.78 (t, J = 6.4 Hz, 2H), 6.90-7.05 (m, 6H), 7.10-7.20 (m, 6H), 7.22 (d, J = 5.2 Hz, 2H), 7.31 (d, J = 5.2 Hz, 2H), 7.62 (d, J = 8.7 Hz, 2H), 7.70-7.95 (m, 8H), 7.98 (d, J = 8.1 Hz, 2H), 8.20 (d, J = 7.5 Hz, 2H), 8.43 (t, J = 8.1 Hz, 1H), 8.52 (d, J = 7.8 Hz, 2H), 8.71 (d, J = 7.8 Hz, 2H), 8.82 (s, 2H), 9.06 (d, J = 8.1 Hz, 2H). MALDI-MS (m/z): 1021.3 for $[M - PF_6]^+$. Anal. Calcd for: $C_{63}H_{47}F_6N_6O_2PRu \cdot H_2O$: C, 63.90; H, 4.17; N, 7.10. Found: C, 63.85; H, 4.22; N, 7.23.

Synthesis of Complex 5(PF_6). This complex was prepared from [Ru(tpy)Cl_3] and ligand **17** as a red solid in 58% yield, using the similar procedure for the synthesis of complex **2**(PF_6). ^{1}H NMR (400 MHz, Acetone-d$_6$): δ 3.82 (s, 6H), 6.79 (t, J = 6.4 Hz, 2H), 6.90-7.05 (m, 6H), 7.10-7.20 (m, 6H), 7.23 (d, J = 5.6 Hz, 2H), 7.32 (d, J = 5.6 Hz, 2H), 7.60 (d, J = 8.8 Hz, 2H), 7.70-7.80 (m, 4H), 7.85 (m, 4H), 7.90 (d, J = 8.4 Hz, 2H), 7.95 (d, J = 8.4 Hz, 2H), 8.00 (d, J = 8.0 Hz, 2H), 8.22 (d, J = 8.0 Hz, 2H), 8.42 (t, J = 8.4 Hz, 1H), 8.53 (d, J = 8.0 Hz, 2H), 8.71 (d, J = 8.4 Hz, 2H), 8.81 (s, 2H), 9.05 (d, J = 8.0 Hz, 2H). MALDI-MS (m/z): 1097.4 for $[M - PF_6]^+$. Anal. Calcd for: $C_{69}H_{51}F_6N_6O_2PRu \cdot 2H_2O$: C, 64.83; H, 4.34; N, 6.57. Found: C, 65.10; H, 4.15; N, 6.61.

Synthesis of Complex 6(PF_6). This complex was prepared from [Ru(tpy)Cl_3] and ligand **18** as a red solid in 60% yield, using the similar procedure for the synthesis of complex **2**(PF_6). ^{1}H NMR (400 MHz, Acetone-d_6): δ 3.82(s, 6H), 6.80 (t, *J* = 6.0 Hz, 2H), 6.95 (d, *J* = 8.8 Hz, 6H), 7.05- 7.20 (m, 6H), 7.23 (d, *J* = 5.6 Hz, 2H), 7.32 (d, *J* = 5.2 Hz, 2H), 7.59 (d, *J* = 8.4 Hz, 2H), 7.75 (m, 4H), 7.80-8.05 (m, 14H), 8.23 (d, *J* = 8.4 Hz, 2H), 8.42 (t, *J* = 8.4 Hz, 1H), 8.54 (d, *J* = 8.0 Hz, 2H), 8.71 (d, *J* = 8.0 Hz, 2H), 8.81 (s, 2H), 9.05 (d, *J* = 8.0 Hz, 2H). MALDI-MS (m/z): 1173.4 for $[M – PF_6]^+$. Anal. Calcd for: $C_{75}H_{55}F_6N_6O_2PRu·2H_2O$: C, 66.51; H, 4.39; N, 6.21. Found: C, 66.92; H, 4.22; N, 6.47.

Table S1. Crystallographic data of compounds **3**(PF_6) and **4**(PF_6).

Compound	**3**(PF_6)	**4**(PF_6)
CCDC No.	1031690	1031691
Empirical formula	C57 H43 F6 N6 O2 P Ru	C63 H47 F6 N6 O2 P Ru
Formula weight	1090.01	1166.10
Temperature	173.1500 K	173.1500 K
Wavelength	0.71073 Å	0.71073 Å
Crystal system	Triclinic	Monoclinic
Space group	P -1	P 1 21/c 1
Unit cell dimensions	a = 14.490(4) Å	a = 51.940(10) Å
	b = 18.730(5) Å	b = 8.791(2) Å
	c = 20.386(7) Å	c = 26.807(7)Å
	α = 86.12(3)°	α = 90°
	β = 81.060(10)°	β = 93.558(4)°
	γ = 81.730(10)°	γ = 90°
Volume	5403(3) $Å^3$	12216(5) $Å^3$
Z	4	8
Calculated density	1.340 mg/m^3	1.268 mg/m^3
Crystal size	0.3 × 0.2 × 0.05 mm^3	0.33 × 0.28 × 0.08 mm^3
final R indices	R1 = 0.0930, wR2 = 0.2003	R1 = 0.1111, wR2 = 0.2823
R indices (all data)	R1 = 0.1039, wR2 = 0.2077	R1 = 0.1291, wR2 = 0.3018

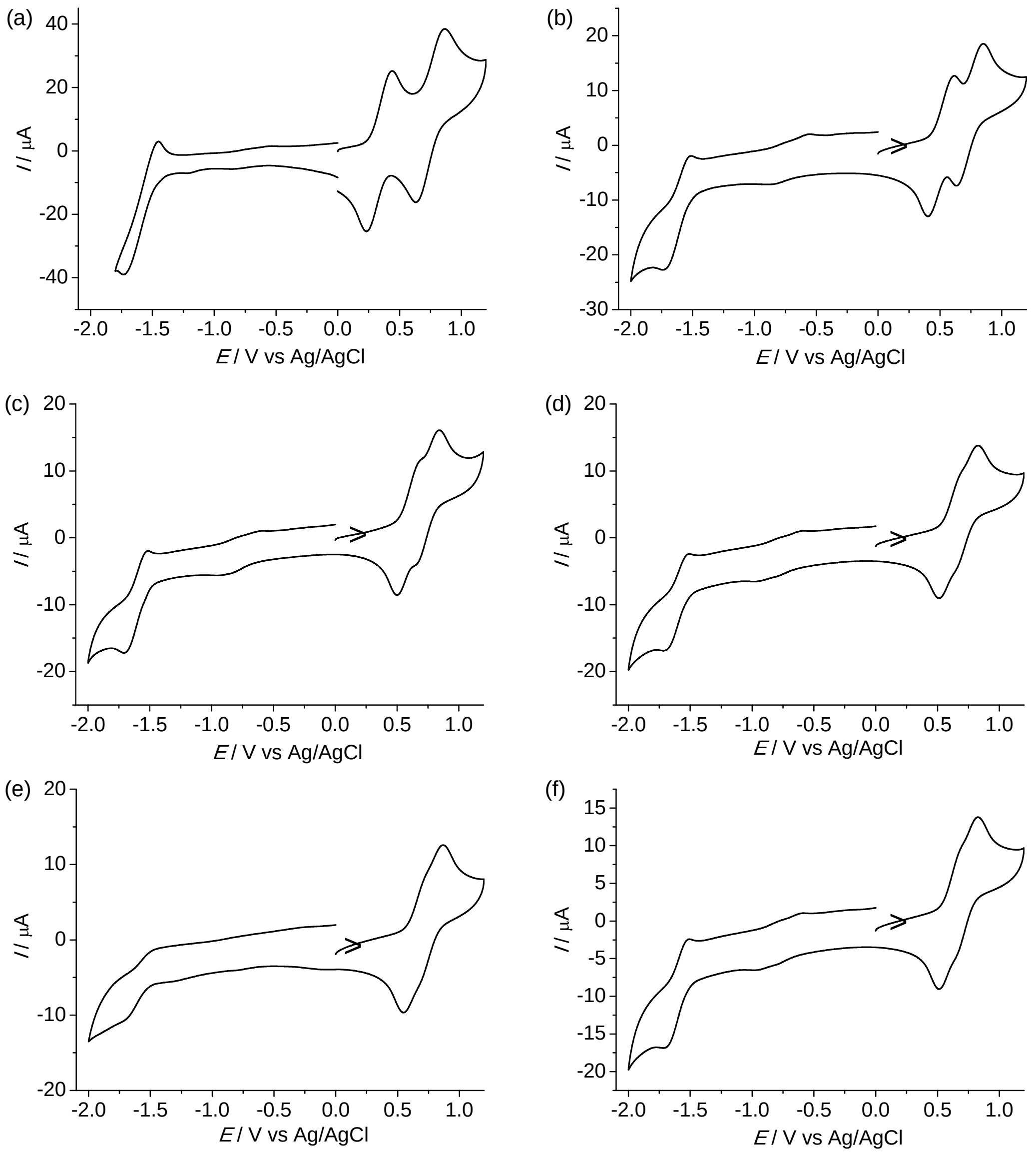

Figure S1. CVs of (a-f) complexes **1** through **6** in 0.1 M Bu_4NClO_4/CH_2Cl_2 at a glassy carbon disk electrode (d = 3 mm) at 100 mV/s.

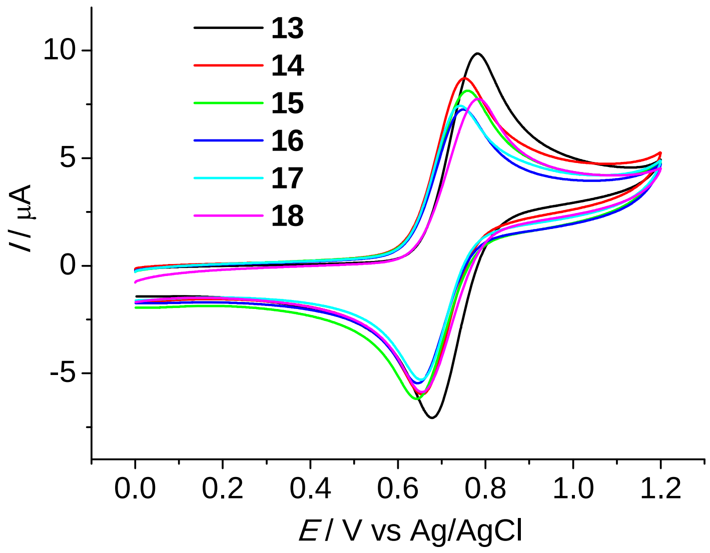

Figure S2. CVs of ligands **13 - 18** in 1:1 mixture of CH_2Cl_2 and CH_3CN containing 0.1 M Bu_4NClO_4 at a Pt disk electrode (d = 2 mm) at 100 mV/s.

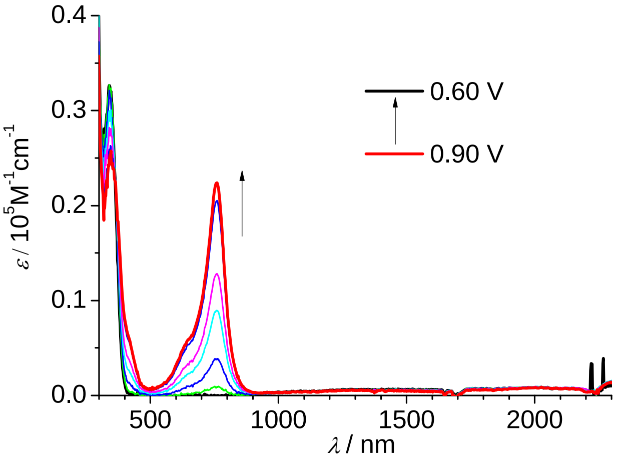

Figure S3. Absorption spectral changes of ligand **14** upon oxidation in 0.1 M Bu_4NClO_4/CH_2Cl_2 by stepwise electrolysis using an ITO glass electrode.

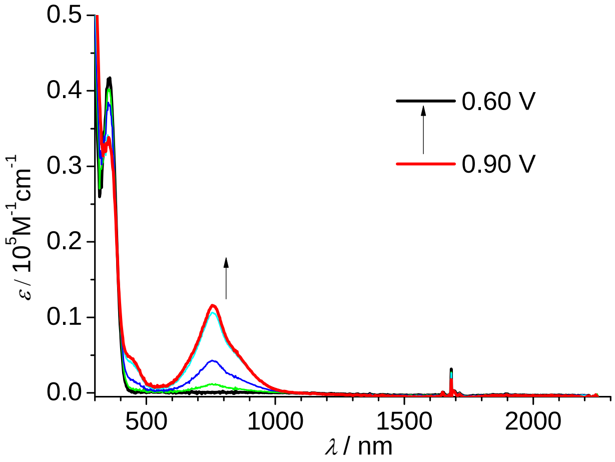

Figure S4. Absorption spectral changes of ligand **16** upon oxidation in 0.1 M Bu_4NClO_4/CH_2Cl_2 by stepwise electrolysis using an ITO glass electrode.

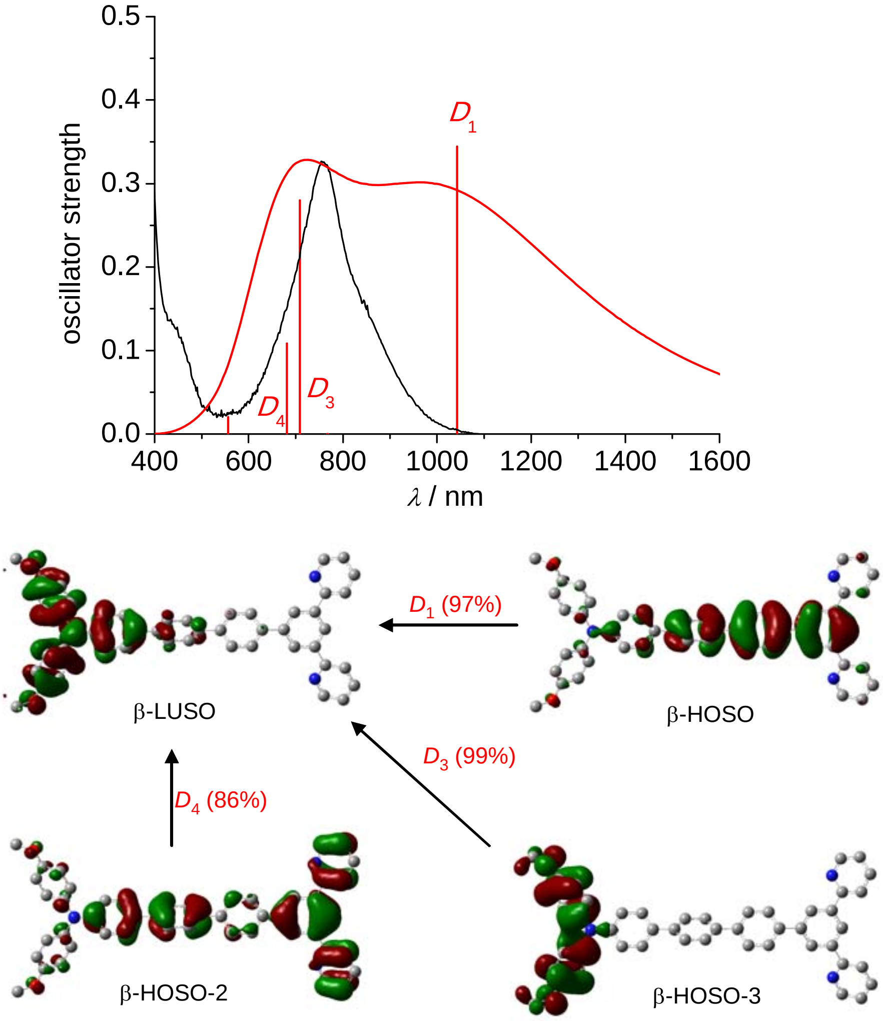

Figure S5. TDDFT-predicted low-energy doublet excitations of **16**$^{+}$ (red curves). The experimental absorption spectrum of **16**$^{+}$ is included for comparison (black curve). The predicted D_1 and D_4 excitations have the character of the bridge-to-aminium charge transfer transitions. The oscillator strength of D_1 is overestimated by TDDFT results. The predicted D_3 excitation is associated with the $N^{\bullet+}$-localized transitions.

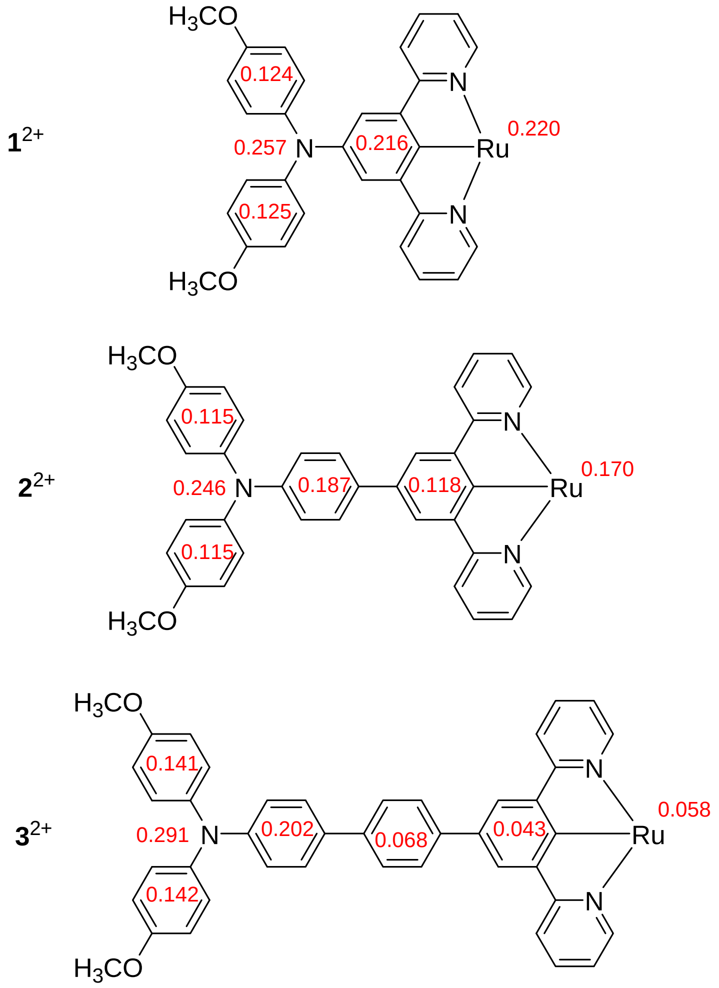

Figure S6. Schematic representations of the DFT-calculated spin distributions of **1**$^{2+}$ - **3**$^{2+}$.

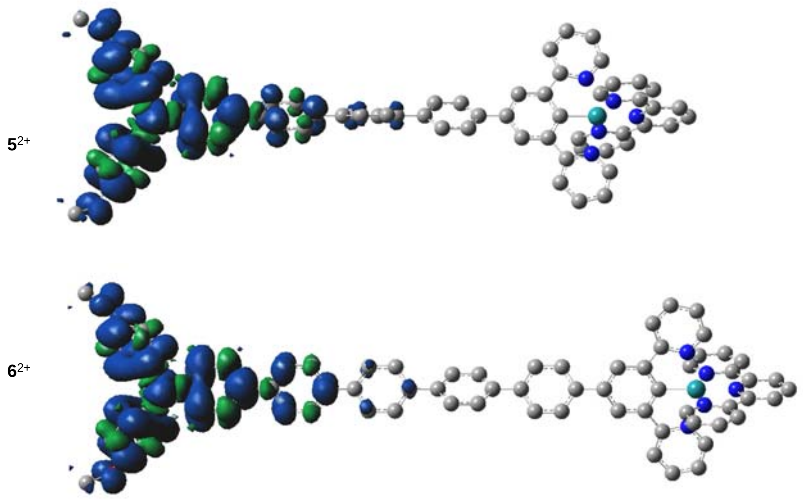

Figure S7. DFT-calculated spin density plot of **5**$^{2+}$ and **6**$^{2+}$.

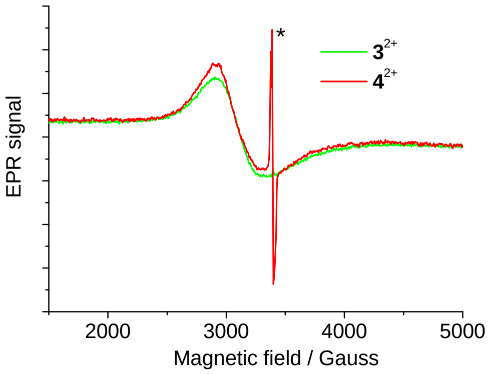

Figure S8. EPR signals of $\mathbf{3}^{2+}$ and $\mathbf{4}^{2+}$. *: impurities.

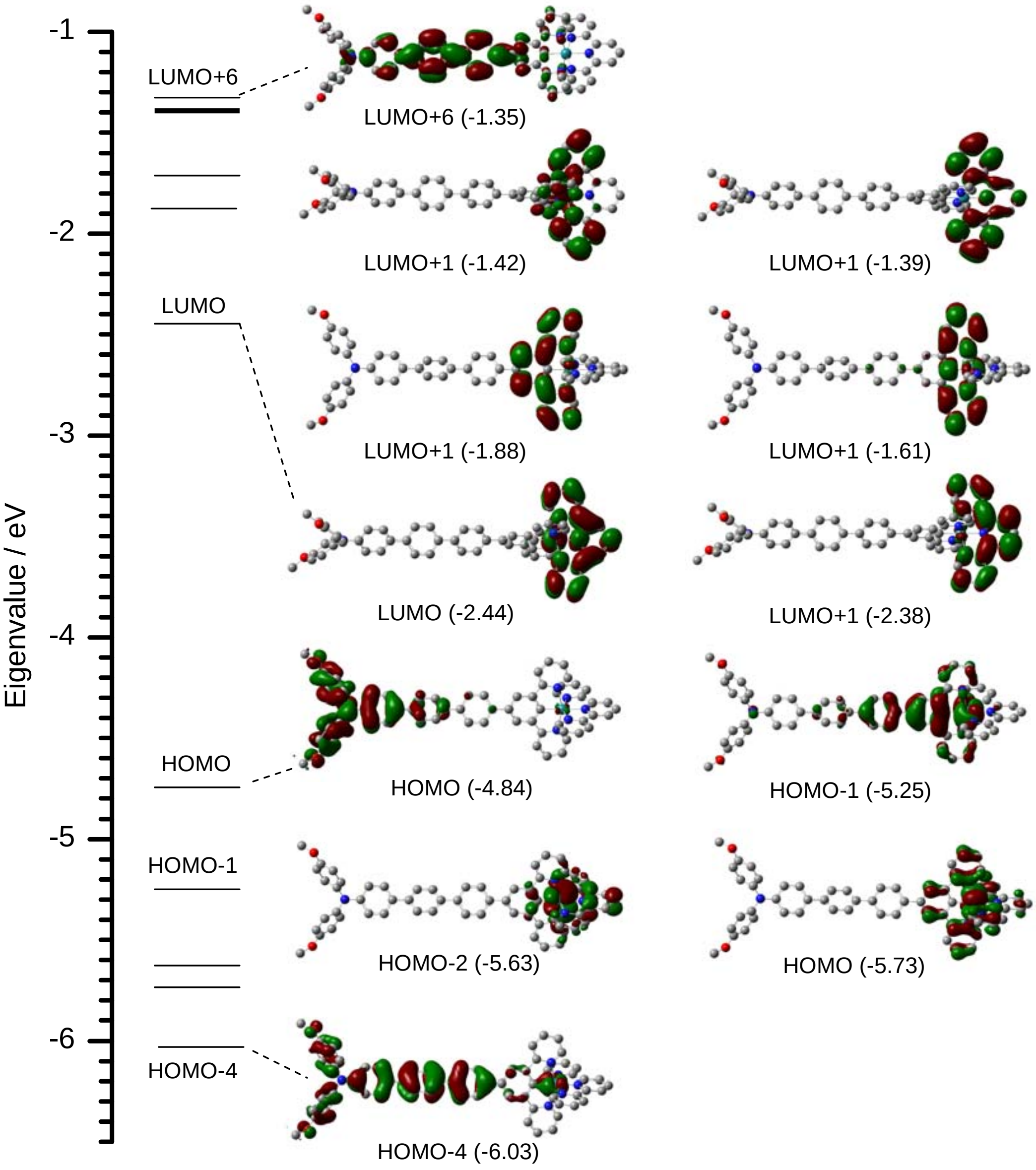

Figure S9. DFT-calculated energy diagram and frontier molecular orbitals of $\mathbf{4}^{2+}$. Eigenvalues in eV are shown in the parenthesis for each energy level.

Cartesian coordinates for the DFT-optimized structure of **1**$^{2+}$:

Charge = 2 multiplicity = 2

Ru	8.77275674	6.08206585	7.24141758
O	12.22006859	-3.38355170	9.09421721
O	6.51505661	0.54763852	15.55690538
N	10.79868374	6.71402324	7.04516929
N	8.63113679	7.82237573	6.06577772
N	6.67924153	6.26929707	6.88092934
N	8.60879233	6.90467236	9.22172691
N	8.99311006	4.56719975	5.73131452
N	9.18025153	1.01759220	10.67875085
C	11.87353597	6.09858502	7.57154163
H	11.67172475	5.20305314	8.14635084
C	13.17163348	6.56917615	7.39806066
H	13.99993902	6.03163023	7.84581547
C	13.37140340	7.72650749	6.64851639
C	12.26567975	8.37105824	6.09957971
H	12.39832036	9.27211104	5.51275178
C	10.98483787	7.85436423	6.30555587
C	9.76305354	8.48333621	5.75374185
C	9.69829987	9.64580490	4.97872994
H	10.59198784	10.19772529	4.71366256
C	8.44689965	10.08993458	4.54738204
C	7.28869962	9.38693171	4.88469542
H	6.32153268	9.73903058	4.54659961
C	7.41234956	8.23100931	5.66211073
C	6.31314559	7.35215051	6.12235058
C	4.97164654	7.58912059	5.81570242
H	4.69723150	8.44846126	5.21568935
C	3.98853966	6.71985890	6.28177692
C	4.37064633	5.62249509	7.05055182
H	3.64299348	4.91706754	7.43577243
C	5.72184119	5.43473427	7.32579669
H	6.06392465	4.59554098	7.91883198
C	8.46060386	8.19931445	9.54907860
H	8.41731193	8.89836754	8.72132775
C	8.36651059	8.63826031	10.86663242
H	8.24858164	9.69583189	11.07454056
C	8.42786473	7.69334903	11.89075191
H	8.35857494	7.99712101	12.93043883
C	8.57973039	6.34881219	11.56207531
H	8.62844656	5.59682090	12.34172903
C	8.66982277	5.96421972	10.22158134
C	8.83020316	4.57980371	9.74243499
C	8.92070231	3.43646284	10.52137316
H	8.89382329	3.47958623	11.60451030
C	9.09030431	2.17060958	9.89633694
C	9.16803103	2.07086479	8.47995207
H	9.26761795	1.08711368	8.03508307
C	9.07740551	3.21695047	7.70495457
C	8.90667208	4.48351217	8.32650622
C	9.11790249	3.29428389	6.23374207
C	9.26988855	2.20451075	5.37203534
H	9.36925761	1.20514774	5.78081990
C	9.29324934	2.40574477	3.99441168
H	9.40992573	1.56256792	3.32103899
C	9.16483099	3.70240660	3.49693401

H	9.17743837	3.90734417	2.43229487
C	9.01805691	4.75084288	4.40057626
H	8.91622272	5.77464049	4.05852265
C	9.97431512	-0.08635622	10.25879922
C	11.26715912	0.11730889	9.75363201
H	11.66994063	1.12318689	9.69518534
C	12.05047268	-0.96009244	9.34927043
H	13.04947535	-0.77446891	8.97413027
C	11.54661826	-2.26705753	9.45332821
C	10.25107905	-2.47284521	9.96713646
H	9.87309738	-3.48771661	10.03401404
C	9.47484287	-1.39882735	10.36575669
H	8.47312457	-1.56543064	10.74737942
C	13.54104556	-3.25127808	8.56470647
H	13.53904537	-2.66163457	7.64101266
H	13.87483300	-4.26648336	8.34920322
H	14.21533464	-2.79085620	9.29553888
C	8.47884042	0.91952588	11.91330772
C	9.12093870	0.39447368	13.05146246
H	10.15987694	0.08836619	12.99133898
C	8.43582964	0.28649847	14.24889622
H	8.92312944	-0.10584458	15.13539623
C	7.09148256	0.69712968	14.34257488
C	6.44581785	1.21736113	13.20881178
H	5.40782693	1.52435317	13.24824369
C	7.13806646	1.32220426	12.00562168
H	6.62710042	1.70019344	11.12609279
C	5.15212920	0.94357808	15.72618410
H	4.48830008	0.36201290	15.07660273
H	5.02336812	2.01282210	15.52393028
H	4.91330849	0.73794175	16.76983065
H	2.94469512	6.89913484	6.04658303
H	8.37371460	10.98921824	3.94515252
H	14.36873555	8.12365385	6.49155929

Cartesian coordinates for the DFT-optimized structure of $\mathbf{2}^{2+}$:

Charge = 2 multiplicity = 2

Ru	0.06422180	-0.03981351	-0.03727625
N	0.50082119	-1.31300424	1.63815736
N	-0.40839550	1.61165257	1.22204506
N	-2.02880496	-0.03139735	-0.02720715
N	-0.43399736	-1.68783539	-1.29146351
N	0.49344023	1.22995257	-1.71729922
C	4.83575183	-0.05909842	-0.06244607
C	4.11342279	0.68742848	-1.02874269
H	4.66541051	1.22525535	-1.79331771
C	2.72136672	0.69491871	-1.02581592
C	2.72562609	-0.79567351	0.92349505
C	4.11767067	-0.79971184	0.91152958
H	4.67336257	-1.34227666	1.67004883
C	1.83541148	1.40540835	-1.96194880
C	2.25890425	2.20757915	-3.02731955
H	3.31967681	2.33961609	-3.21013727
C	1.32255648	2.83171607	-3.84572217
H	1.64839863	3.45425569	-4.67315009
C	-0.03626444	2.64535721	-3.58623638
H	-0.80170003	3.11174845	-4.19647456
C	-0.40264342	1.83855855	-2.51403446
H	-1.44573726	1.66527254	-2.27335572
C	1.84383949	-1.49892327	1.86899197
C	2.27200951	-2.30411782	2.93020849
H	3.33355021	-2.44425826	3.10223492
C	1.33928107	-2.92086260	3.75829263
H	1.66874305	-3.54573701	4.58252095
C	-0.02064501	-2.72410365	3.51264000
H	-0.78336653	-3.18452202	4.13076079
C	-0.39174725	-1.91458410	2.44411907
H	-1.43588248	-1.73318488	2.21414517
C	0.43327522	-2.50527640	-1.91682427
H	1.48264175	-2.29059472	-1.75447983
C	0.02589203	-3.56274755	-2.72435147
H	0.77030618	-4.18894449	-3.20338249
C	-1.33863584	-3.78881290	-2.89639920
H	-1.69439710	-4.60328847	-3.51867347
C	-2.24505139	-2.94898839	-2.25460700
H	-3.31045784	-3.10566360	-2.37503932
C	-1.77972635	-1.90253798	-1.45474446
C	-2.68243129	-0.96999917	-0.74226715
C	-4.08083925	-0.98668903	-0.75337762
H	-4.63114161	-1.72971010	-1.31816542
C	-4.76575087	-0.01872338	-0.01590507
C	-4.06587051	0.94282333	0.71587045
H	-4.60460856	1.69103879	1.28494757
C	-2.66781622	0.91315911	0.69322091
C	-1.75070877	1.83747435	1.39798845
C	-2.19977408	2.88687875	2.20321968
H	-3.26265743	3.05224743	2.33385939
C	-1.28044400	3.71843844	2.83736462
H	-1.62363897	4.53513778	3.46376685
C	0.08049372	3.48120274	2.65235206
H	0.83454103	4.10067312	3.12501553
C	0.47146879	2.42117676	1.84005910

H	1.51743060	2.19782424	1.66771492
C	6.30245193	-0.06513098	-0.07042718
C	7.04238592	-1.15329005	0.45493085
C	7.04537932	1.01692388	-0.60410107
C	8.42407100	-1.16517268	0.45275736
H	6.52177425	-2.02908390	0.82681109
C	8.42703270	1.01716867	-0.61804990
H	6.52786715	1.89710762	-0.96988080
C	9.14786234	-0.07711858	-0.08700383
H	8.95701679	-2.03212662	0.82637343
H	8.96287547	1.87960665	-0.99793765
C	11.27021131	-0.71694240	0.94815896
C	10.87252677	-0.55699241	2.29178628
C	12.40041950	-1.49783108	0.65460318
C	11.58583093	-1.16965290	3.30551874
H	10.01890137	0.06768052	2.53176481
C	13.12015454	-2.11488001	1.67137800
H	12.70405146	-1.63844660	-0.37712731
C	12.71706943	-1.95653286	3.00896525
H	11.29880788	-1.04175846	4.34394517
H	13.97937736	-2.72228986	1.41503582
C	11.26289257	0.54314963	-1.14884489
C	12.40529148	1.31165604	-0.86997419
C	10.84567806	0.38839824	-2.48717481
C	13.11808032	1.92142306	-1.89595992
H	12.72408404	1.44841236	0.15769093
C	11.55201825	0.99391583	-3.51002798
H	9.98226839	-0.22686924	-2.71616195
C	12.69559187	1.76825521	-3.22815208
H	13.98713436	2.51940691	-1.65088870
H	11.24981406	0.86978854	-4.54459537
N	10.54717876	-0.08332068	-0.09573444
O	13.34164183	-2.50852004	4.07101555
O	13.31209030	2.31410223	-4.29805368
C	14.50908775	-3.30545978	3.85088467
H	14.83706509	-3.62479477	4.84015282
H	14.27670422	-4.18435702	3.23940168
H	15.30101241	-2.71929242	3.37199423
C	14.49085194	3.09829115	-4.09269032
H	14.80941749	3.41468540	-5.08596640
H	14.27584161	3.97921180	-3.47778482
H	15.28250555	2.50331854	-3.62431695
H	-5.85054239	-0.01366748	-0.01153424
C	2.01496270	-0.04750388	-0.04741896

Cartesian coordinates for the DFT-optimized structure of **3**$^{2+}$:

Charge = 2 multiplicity = 2

Ru	0.05438470	-0.04612474	-0.03869807
N	0.50521549	-1.38740351	1.57786054
N	-0.40836978	1.55125990	1.28768103
N	-2.02492232	-0.04003449	-0.02951531
N	-0.42949516	-1.64109155	-1.36047774
N	0.49743883	1.29273821	-1.65946603
C	4.84177350	-0.06114666	-0.06266621
C	4.12464991	0.72199734	-0.99365861
H	4.68096717	1.29577794	-1.73002122
C	2.72639201	0.72944563	-0.99007318
C	2.73086790	-0.83762697	0.88657565
C	4.12911801	-0.83949559	0.87574837
H	4.68915422	-1.41718568	1.60621445
C	1.84142052	1.47826335	-1.89363672
C	2.26481127	2.32627040	-2.92498397
H	3.32615690	2.46519836	-3.09981306
C	1.33078325	2.98416714	-3.71765366
H	1.65805369	3.64106586	-4.51771185
C	-0.02944059	2.78700977	-3.46880640
H	-0.79389359	3.27870786	-4.06031009
C	-0.39669077	1.93581965	-2.43280247
H	-1.44053511	1.75246215	-2.20198136
C	1.85028494	-1.58087754	1.79898993
C	2.27865050	-2.43106713	2.82647984
H	3.34080875	-2.57621087	2.99103492
C	1.34848311	-3.08314867	3.62843660
H	1.67960102	-3.74172311	4.42552886
C	-0.01290150	-2.87799877	3.39274540
H	-0.77446554	-3.36497421	3.99183317
C	-0.38515985	-2.02493056	2.36007905
H	-1.43009661	-1.83537354	2.13941910
C	0.43816745	-2.43333867	-2.01752832
H	1.48718053	-2.22233879	-1.84641228
C	0.03264068	-3.45985419	-2.86462431
H	0.77771654	-4.06651056	-3.36744649
C	-1.33205707	-3.68180446	-3.04490190
H	-1.68658561	-4.47302779	-3.69728149
C	-2.23936928	-2.86842720	-2.37165269
H	-3.30463532	-3.02271434	-2.49718321
C	-1.77568252	-1.85176503	-1.53241072
C	-2.67935330	-0.94951431	-0.78390362
C	-4.07768195	-0.96588348	-0.79615135
H	-4.62715147	-1.68464221	-1.39256787
C	-4.76459858	-0.03075724	-0.01883957
C	-4.06533336	0.89967430	0.75307340
H	-4.60524199	1.62226538	1.35357910
C	-2.66728453	0.87385330	0.72990867
C	-1.75173083	1.77012186	1.47113010
C	-2.20198950	2.78894735	2.31504069
H	-3.26517482	2.94970982	2.44977420
C	-1.28399112	3.59615028	2.98119862
H	-1.62812921	4.38900562	3.63715142
C	0.07774100	3.36590282	2.78924175
H	0.83077190	3.96752945	3.28622247
C	0.46971881	2.33757120	1.93797249

H	1.51590478	2.12013861	1.75780295
C	6.32248626	-0.06620831	-0.07054433
C	7.05448011	-1.22097773	0.27095592
C	7.05866469	1.08350759	-0.42000733
C	8.44374258	-1.22518280	0.26929056
H	6.52747847	-2.14014598	0.50825759
C	8.44785397	1.07805150	-0.43362430
H	6.53546396	2.00630368	-0.65158764
C	9.17782330	-0.07611340	-0.08622857
H	8.96478389	-2.14810023	0.50497717
H	8.97265048	1.99736796	-0.67498278
H	-5.84943038	-0.02705913	-0.01467367
C	2.02152841	-0.05179912	-0.04815300
C	10.65300891	-0.08119748	-0.09436715
C	11.38295299	0.74110277	-0.98035118
C	11.38701866	-0.90850060	0.78356992
C	12.76837248	0.73884450	-0.99707331
H	10.85567861	1.38243925	-1.67865982
C	12.77252387	-0.91540710	0.78527566
H	10.86308942	-1.54635407	1.48756217
C	13.48223518	-0.09053793	-0.10965145
H	13.30494365	1.38933731	-1.67880895
H	13.31220233	-1.56942335	1.46116463
C	15.60600218	-0.17157029	1.09969180
C	15.14662071	0.52560979	2.24025267
C	16.78154198	-0.94209026	1.19042159
C	15.84776776	0.45151475	3.42656914
H	14.25799601	1.14330090	2.17393351
C	17.48570724	-1.02105218	2.38340982
H	17.12438824	-1.50429712	0.32899095
C	17.02521794	-0.32247769	3.51557217
H	15.51766059	0.99751740	4.30380557
H	18.37574591	-1.63605187	2.43116568
C	15.59412300	-0.02178534	-1.34042327
C	16.77303757	0.74206086	-1.44292468
C	15.11907566	-0.71586000	-2.47644230
C	17.46544044	0.81745559	-2.64301295
H	17.12777863	1.30202153	-0.58485034
C	15.80846116	-0.64528140	-3.66983774
H	14.22767066	-1.32850652	-2.40119424
C	16.98936491	0.12197735	-3.77063704
H	18.35844218	1.42738872	-2.69965377
H	15.46628579	-1.18903492	-4.54384026
O	17.62976040	-0.32782109	4.71741700
O	17.58158548	0.12422290	-4.97861019
C	18.84021848	-1.07515793	4.88751664
H	19.13796308	-0.91617957	5.92364119
H	18.66869489	-2.14249255	4.71248408
H	19.62407281	-0.70749918	4.21705495
C	18.79460949	0.86447596	-5.16089793
H	19.08118580	0.70348594	-6.19985885
H	18.63100519	1.93283759	-4.98455737
H	19.58289923	0.49249323	-4.49807104
N	14.89505519	-0.09479084	-0.11682762

Cartesian coordinates for the DFT-optimized structure of $\mathbf{4}^{2+}$:

Charge = 2 multiplicity = 2

Ru	0.01053474	-0.05517749	-0.03880772
N	0.46923863	-1.53091174	1.45436039
N	-0.45795966	1.41758434	1.42123720
N	-2.06502812	-0.06056827	-0.03146157
N	-0.46062047	-1.53049814	-1.49539315
N	0.45040186	1.42300427	-1.53534139
C	4.80152935	-0.04344997	-0.05749524
C	4.08154423	0.81268314	-0.91748223
H	4.63628863	1.45250933	-1.59893339
C	2.68186062	0.81321248	-0.91452026
C	2.69285822	-0.90995613	0.81654563
C	4.09253453	-0.90290498	0.80827174
H	4.65582066	-1.54023664	1.48504018
C	1.79438130	1.63435294	-1.74955102
C	2.21517330	2.57253324	-2.70134704
H	3.27619954	2.73068850	-2.86097550
C	1.27968096	3.29420695	-3.43406917
H	1.60527655	4.02115079	-4.17186561
C	-0.08017404	3.06984979	-3.20634484
H	-0.84588871	3.60895696	-3.75329117
C	-0.44522544	2.12849627	-2.25086752
H	-1.48908908	1.92163791	-2.04051578
C	1.81586886	-1.73548945	1.65836484
C	2.24860640	-2.67143871	2.60697861
H	3.31161024	-2.82416587	2.75847758
C	1.32237763	-3.39773008	3.34686957
H	1.65722684	-4.12294588	4.08221998
C	-0.04028041	-3.18022409	3.12949911
H	-0.79908323	-3.72310659	3.68231142
C	-0.41734366	-2.24081984	2.17675852
H	-1.46383708	-2.03929583	1.97442571
C	0.41209277	-2.25684716	-2.21878170
H	1.45992846	-2.05717771	-2.02742268
C	0.01311484	-3.20564292	-3.15486403
H	0.76199367	-3.76114103	-3.70863573
C	-1.35022307	-3.41766968	-3.35670030
H	-1.69974987	-4.14905606	-4.07799601
C	-2.26271126	-2.67283094	-2.61479109
H	-3.32685103	-2.82141579	-2.75549164
C	-1.80574791	-1.73237960	-1.68734388
C	-2.71475383	-0.90453644	-0.86297190
C	-4.11295631	-0.92738499	-0.87726096
H	-4.65876982	-1.59430326	-1.53426090
C	-4.80547015	-0.06761332	-0.02183329
C	-4.11139257	0.79577376	0.82867284
H	-4.65598165	1.45983822	1.48956542
C	-2.71322823	0.78014632	0.80452273
C	-1.80272805	1.61269865	1.62247184
C	-2.25800847	2.55093284	2.55298803
H	-3.32188781	2.69425974	2.70094560
C	-1.34417386	3.30038239	3.28856912
H	-1.69238835	4.03008726	4.01219916
C	0.01878945	3.09511310	3.07742740
H	0.76866332	3.65436211	3.62605359
C	0.41606668	2.14829505	2.13862355

H	1.46354370	1.95389323	1.94001098
C	6.28561769	-0.04011573	-0.06394790
C	7.02158688	-1.22310605	0.13476212
C	7.01436313	1.14617956	-0.26946968
C	8.41298008	-1.22023741	0.12989758
H	6.49752947	-2.16348128	0.27944471
C	8.40572517	1.14952605	-0.27821084
H	6.48473597	2.08420041	-0.40903202
C	9.14005158	-0.03375090	-0.07784162
H	8.94165059	-2.15271525	0.30509801
H	8.92846889	2.08434764	-0.45859974
H	-5.89030694	-0.07043970	-0.01798767
C	1.98034669	-0.05004733	-0.04616920
C	10.62130665	-0.03051960	-0.08555402
C	11.35241846	1.07918333	0.37998911
C	11.35225860	-1.13702234	-0.55889082
C	12.74225906	1.08493584	0.36886101
H	10.82624816	1.93747391	0.78651121
C	12.74214195	-1.13665035	-0.56270024
H	10.82553715	-1.99760833	-0.95979815
H	13.26685141	1.94744597	0.76862200
H	13.26619100	-1.99685403	-0.96810695
C	13.47205365	-0.02424420	-0.10085314
C	14.94898201	-0.02102486	-0.10886230
C	15.67574938	1.17499321	-0.29144186
C	15.68280321	-1.21391379	0.06578299
C	17.06174588	1.18572448	-0.30393477
H	15.14603380	2.10997516	-0.44027540
C	17.06886501	-1.21867525	0.06324986
H	15.15877628	-2.15115763	0.22037275
C	17.77522969	-0.01494483	-0.12410997
H	17.59791779	2.11953353	-0.43205343
H	17.61037436	-2.15018473	0.18550859
C	19.89987598	-0.81795704	0.78184411
C	19.43572133	-0.97298177	2.10840617
C	21.07885521	-1.47792849	0.38196181
C	20.13668010	-1.75933545	2.99918482
H	18.54462595	-0.44787664	2.43354024
C	21.78189825	-2.27339654	1.27469833
H	21.42305035	-1.38979895	-0.64244102
C	21.31730417	-2.42122731	2.59580006
H	19.80415776	-1.87083276	4.02560211
H	22.67329013	-2.78667861	0.93605251
C	19.88714376	0.79664368	-1.05213497
C	21.06771963	1.46118514	-0.66468756
C	19.40861893	0.94964759	-2.37382371
C	21.75867562	2.25868100	-1.56500698
H	21.42281963	1.37491636	0.35615963
C	20.09755172	1.73804226	-3.27215113
H	18.51604619	0.42127450	-2.68947901
C	21.28007491	2.40409154	-2.88135994
H	22.65165905	2.77532199	-1.23580200
H	19.75402065	1.84790691	-4.29511530
N	19.18997263	-0.01197272	-0.13143686
O	21.91958292	-3.16428323	3.54068481
O	21.87019930	3.14848965	-3.83282768
C	23.13281511	-3.85613706	3.21982533
H	23.42738252	-4.36779827	4.13568881

H	22.96545725	-4.59001105	2.42455757
H	23.91644544	-3.15225421	2.92087060
C	23.08472269	3.84399808	-3.52500382
H	23.36848393	4.35564500	-4.44428176
H	22.92348788	4.57814254	-2.72871758
H	23.87332665	3.14255647	-3.23345323

Cartesian coordinates for the DFT-optimized structure of $\mathbf{5}^{2+}$:

Charge = 2 multiplicity = 2

Ru	-0.00123456	-0.04100743	-0.02876368
N	0.46414411	-1.84223632	1.04511848
N	-0.45155947	1.02055063	1.75765809
N	-2.07760522	-0.04373930	-0.00342413
N	-0.49223181	-1.10449005	-1.80304143
N	0.43429828	1.76182859	-1.11325076
C	4.79090130	-0.03299238	-0.08201038
C	4.06793512	1.00958700	-0.69882756
H	4.62069494	1.79650202	-1.20529587
C	2.66798954	1.01253670	-0.68386069
C	2.68520297	-1.08659610	0.56477690
C	4.08506972	-1.07845165	0.54965412
H	4.65149126	-1.86307706	1.04444072
C	1.77724215	2.01767378	-1.27961557
C	2.19319312	3.16386312	-1.96998097
H	3.25340405	3.35599050	-2.09393086
C	1.25427529	4.04717224	-2.49078124
H	1.57652488	4.93513062	-3.02604946
C	-0.10459381	3.77476807	-2.31477046
H	-0.87301035	4.43428700	-2.70292073
C	-0.46460977	2.62479347	-1.62179493
H	-1.50692481	2.37177617	-1.46021886
C	1.81123183	-2.09421168	1.18080530
C	2.24603636	-3.23875284	1.86221342
H	3.30931225	-3.42797337	1.96206467
C	1.32170164	-4.12424447	2.40494871
H	1.65858076	-5.01093296	2.93326893
C	-0.04160020	-3.85572462	2.25998885
H	-0.79905768	-4.51709087	2.66611843
C	-0.42056300	-2.70722342	1.57471717
H	-1.46695983	-2.45690224	1.43700397
C	0.37098420	-1.62825050	-2.69343161
H	1.42097420	-1.48413602	-2.46734468
C	-0.03980470	-2.31169845	-3.83354233
H	0.70203726	-2.71250141	-4.51551058
C	-1.40582077	-2.46314053	-4.06953876
H	-1.76479079	-2.98973647	-4.94773035
C	-2.30863402	-1.92530699	-3.15642438
H	-3.37469986	-2.03055863	-3.32025976
C	-1.83974105	-1.24864779	-2.02702082
C	-2.73806573	-0.65170960	-1.01348789
C	-4.13641884	-0.66741575	-1.01972929
H	-4.69055195	-1.14883594	-1.81691557
C	-4.81756944	-0.04575407	0.02923591
C	-4.11253978	0.57676553	1.06175971
H	-4.64813927	1.05753113	1.87189377
C	-2.71473543	0.56299481	1.02224721
C	-1.79358312	1.16090147	2.01450783
C	-2.23663708	1.83409480	3.15634310
H	-3.29869586	1.93603605	3.34636704
C	-1.31325417	2.37233753	4.04840115
H	-1.65209387	2.89617329	4.93619069
C	0.04700704	2.22487682	3.77898817
H	0.80417245	2.62620776	4.44357889
C	0.43170326	1.54477891	2.62781802

H	1.47619692	1.40386336	2.37580643
C	6.27574668	-0.02966693	-0.09571426
C	7.01010574	-1.22566084	-0.19663444
C	7.00647389	1.16949596	-0.00658216
C	8.40197907	-1.22336965	-0.20341914
H	6.48498897	-2.17244902	-0.28598913
C	8.39830065	1.17294222	-0.02001438
H	6.47883528	2.11411558	0.09058881
C	9.13107895	-0.02372899	-0.11641296
H	8.92986523	-2.17081095	-0.26352137
H	8.92311700	2.12253073	0.03258573
H	-5.90236646	-0.04646691	0.04210125
C	1.96989400	-0.03822242	-0.05186798
C	10.61396341	-0.02090167	-0.12448530
C	11.34642902	0.92641369	0.61439819
C	11.34257576	-0.96588397	-0.87012574
C	12.73781635	0.93019924	0.60667905
H	10.82135565	1.65068152	1.23016081
C	12.73401395	-0.96595209	-0.87431771
H	10.81416413	-1.69170432	-1.48119001
H	13.26637933	1.65736651	1.21618585
H	13.25931492	-1.69185412	-1.48815611
C	13.46521213	-0.01709921	-0.13667892
C	14.94682048	-0.01592604	-0.14150054
C	15.67575187	1.18604401	-0.07186819
C	15.67702967	-1.21689576	-0.21486676
C	17.06596602	1.18825444	-0.07859008
H	15.14732335	2.13363477	-0.02932529
C	17.06726483	-1.21729350	-0.21410317
H	15.14964231	-2.16516931	-0.25500303
C	17.79553681	-0.01405482	-0.14761203
H	17.59037892	2.13629117	-0.00646382
H	17.59261586	-2.16464702	-0.28838485
C	19.27307045	-0.01305763	-0.14948457
C	20.00409765	-1.06758643	0.43781847
C	20.00135296	1.04264008	-0.73809730
C	21.39032047	-1.07163061	0.44444223
H	19.47766069	-1.89048146	0.90967162
C	21.38756139	1.04919594	-0.74670714
H	19.47273479	1.86461922	-1.20911570
C	22.09832100	-0.01050469	-0.15150397
H	21.93051741	-1.89979329	0.88969608
H	21.92561961	1.87835130	-1.19269696
C	24.21741558	-0.43101710	0.99401093
C	23.74342146	-0.10141695	2.28482198
C	25.39964206	-1.18718210	0.86560641
C	24.43840259	-0.51313940	3.40304594
H	22.84943450	0.50220771	2.39382043
C	26.09626591	-1.60724979	1.98894884
H	25.75116191	-1.47254377	-0.11958529
C	25.62232471	-1.27222273	3.27203055
H	24.09830228	-0.24982627	4.39882554
H	26.98993950	-2.20580175	1.86304139
C	24.21575084	0.41462515	-1.29852074
C	25.39643212	1.17331747	-1.17078894
C	23.74156548	0.08424941	-2.58905874
C	26.09134568	1.59512443	-2.29453417
H	25.74804585	1.45924734	-0.18579526

C	24.43486388	0.49770870	-3.70769044
H	22.84880176	-0.52127751	-2.69754376
C	25.61721217	1.25935514	-3.57734663
H	26.98380401	2.19559188	-2.16913550
H	24.09463053	0.23386912	-4.70328475
N	23.51378484	-0.00902529	-0.15204773
O	26.21766949	-1.62546577	4.42439573
O	26.21095974	1.61419539	-4.73004354
C	27.43039939	-2.38789543	4.38154821
H	27.71597359	-2.53948312	5.42213324
H	27.26546426	-3.35686228	3.89893121
H	28.21945876	-1.83836811	3.85777299
C	27.42191900	2.37946802	-4.68780892
H	27.70653778	2.53185428	-5.72853830
H	27.25497083	3.34798033	-4.20496879
H	28.21256395	1.83173877	-4.16454690

Cartesian coordinates for the DFT-optimized structure of $\mathbf{6}^{2+}$:

Charge = 2 multiplicity = 2

Ru	-0.00781551	-0.03895686	-0.03534754
N	0.45035844	-1.65244236	1.30720799
N	-0.47390054	1.28985661	1.55771841
N	-2.08372036	-0.04188230	-0.02731415
N	-0.48239789	-1.36901244	-1.62485248
N	0.43580804	1.57560708	-1.38154393
C	4.78438436	-0.03275211	-0.05382522
C	4.06666887	0.90707929	-0.82243462
H	4.62409238	1.61250458	-1.43337134
C	2.66641412	0.90814032	-0.82083176
C	2.67485646	-0.97886203	0.72985342
C	4.07507877	-0.97430832	0.72045374
H	4.63906415	-1.67840618	1.32687058
C	1.77999824	1.80748415	-1.57156118
C	2.20146670	2.83503574	-2.42591442
H	3.26269249	3.00860414	-2.56745925
C	1.26661356	3.62483891	-3.08551409
H	1.59289915	4.42128065	-3.74737011
C	-0.09361894	3.37818487	-2.88365372
H	-0.85888521	3.96790300	-3.37630648
C	-0.45919801	2.34713536	-2.02606118
H	-1.50282637	2.11849313	-1.83834547
C	1.79656273	-1.88058503	1.48725489
C	2.22720892	-2.90702859	2.33835981
H	3.28993436	-3.07758467	2.47209657
C	1.29945455	-3.69955513	3.00467745
H	1.63282318	-4.49514327	3.66402469
C	-0.06291467	-3.45677864	2.81279668
H	-0.82285495	-4.04877459	3.31093321
C	-0.43771198	-2.42664244	1.95809403
H	-1.48334586	-2.20100895	1.77803732
C	0.38855307	-2.02506098	-2.41449171
H	1.43660757	-1.84672106	-2.20433586
C	-0.01256354	-2.87938002	-3.43666723
H	0.73505972	-3.38087964	-4.04133434
C	-1.37647135	-3.06854779	-3.65714199
H	-1.72758942	-3.72678423	-4.44506414
C	-2.28719786	-2.39669958	-2.84654445
H	-3.35184761	-2.52920182	-2.99956214
C	-1.82790006	-1.54988844	-1.83395729
C	-2.73511834	-0.80307779	-0.93413946
C	-4.13333974	-0.82270392	-0.94947300
H	-4.67993371	-1.42449833	-1.66601897
C	-4.82414829	-0.04597686	-0.01660739
C	-4.12839526	0.73283032	0.91083473
H	-4.67120170	1.33296954	1.63163911
C	-2.73028083	0.71739125	0.88457137
C	-1.81825630	1.46690133	1.77725337
C	-2.27207952	2.31253601	2.79328311
H	-3.33586942	2.44198414	2.95463823
C	-1.35700407	2.98713008	3.59667044
H	-1.70388943	3.64446669	4.38721340
C	0.00568869	2.80185610	3.36561729
H	0.75654651	3.30560191	3.96438115
C	0.40128448	1.94852989	2.34046513

H	1.44816853	1.77318317	2.12211899
C	6.26947487	-0.03113705	-0.06007492
C	7.00206860	-1.23177314	-0.03475891
C	6.99924152	1.17106437	-0.09186063
C	8.39425409	-1.23054168	-0.03999145
H	6.47485375	-2.18174664	-0.02627425
C	8.39141807	1.17280385	-0.09953586
H	6.46992584	2.11990078	-0.09550313
C	9.12287194	-0.02809401	-0.07325940
H	8.92349198	-2.17859381	-0.00375275
H	8.91828572	2.12197682	-0.14069923
H	-5.90900198	-0.04761339	-0.01235955
C	1.96417012	-0.03630498	-0.04278726
C	10.60602141	-0.02648852	-0.08081576
C	11.33846342	0.96777075	0.59254865
C	11.33352599	-1.01904976	-0.76200013
C	12.73023049	0.96924630	0.58549207
H	10.81265243	1.73185709	1.15778930
C	12.72528237	-1.01704004	-0.77024979
H	10.80345602	-1.78443174	-1.32148000
H	13.25884226	1.73399137	1.14701815
H	13.24959732	-1.78042805	-1.33761747
C	13.45792292	-0.02292226	-0.09647820
C	14.94084596	-0.02077838	-0.10507306
C	15.66964596	1.18256350	-0.09132731
C	15.67303507	-1.22192701	-0.12753477
C	17.06093063	1.18498368	-0.09899374
H	15.14160898	2.13155302	-0.09071411
C	17.06432705	-1.22019245	-0.13696684
H	15.14784306	-2.17248092	-0.12172259
C	17.79254675	-0.01649891	-0.12256729
H	17.58550097	2.13545929	-0.06913256
H	17.59133467	-2.16910132	-0.17326138
C	19.27418569	-0.01416185	-0.13215768
C	20.01048148	-1.03578927	0.49749119
C	19.99876005	1.00988045	-0.77144547
C	21.40038823	-1.03680360	0.48488692
H	19.48893619	-1.82350032	1.03233339
C	21.38870008	1.01552300	-0.77727727
H	19.46752827	1.79584479	-1.29928858
C	22.12377305	-0.00944518	-0.15102165
H	21.93015726	-1.82512073	1.01103046
H	21.90878225	1.80558808	-1.31040234
C	23.59989500	-0.00717165	-0.16062675
C	24.32890386	1.20178392	-0.16235760
C	24.33239416	-1.21399021	-0.16812905
C	25.71448956	1.21205345	-0.17250219
H	23.80114195	2.14832369	-0.11306848
C	25.71799599	-1.22024448	-0.17510205
H	23.80681124	-2.16204866	-0.21093499
C	26.42736153	-0.00306576	-0.17808789
H	26.25143751	2.15339774	-0.13496687
H	26.25716718	-2.16002916	-0.21920286
N	27.84047371	-0.00115095	-0.18636166
C	28.54083900	0.95907878	-0.94538588
C	29.72018305	1.54145181	-0.43980610
C	28.06954242	1.34667541	-2.22083909
C	30.41684813	2.48529269	-1.17969034

H	30.07006658	1.27449811	0.55117207
C	28.76421160	2.28147108	-2.96043877
H	27.17839529	0.88513342	-2.63114756
C	29.94588763	2.86415310	-2.45152880
H	31.30841411	2.93284815	-0.75851885
H	28.42611930	2.57248950	-3.94916785
C	28.55183118	-0.95964259	0.56477377
C	28.09683056	-1.34686749	1.84620712
C	29.72573169	-1.54060999	0.04522925
C	28.80207471	-2.27981070	2.57811809
H	27.21000638	-0.88641253	2.26697062
C	30.43301280	-2.48260730	0.77741193
H	30.06291655	-1.27418490	-0.95030124
C	29.97843605	-2.86094593	2.05534391
H	28.47663626	-2.57041433	3.57120661
H	31.32007956	-2.92899382	0.34559286
O	30.54319862	3.76414730	-3.25191153
O	30.58718102	-3.75896775	2.84933289
C	31.75914501	4.38731612	-2.81945538
H	32.04949472	5.05387690	-3.63108003
H	31.59700134	4.96761036	-1.90513530
H	32.54327139	3.64093259	-2.65499903
C	31.79927215	-4.37980414	2.40299762
H	32.10089784	-5.04479858	3.21179203
H	31.62754408	-4.96150717	1.49131977
H	32.57962539	-3.63176756	2.22838011

^1H NMR and ^{13}C NMR spectra of boronic acid **7**:

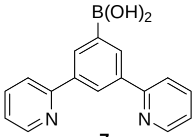

7

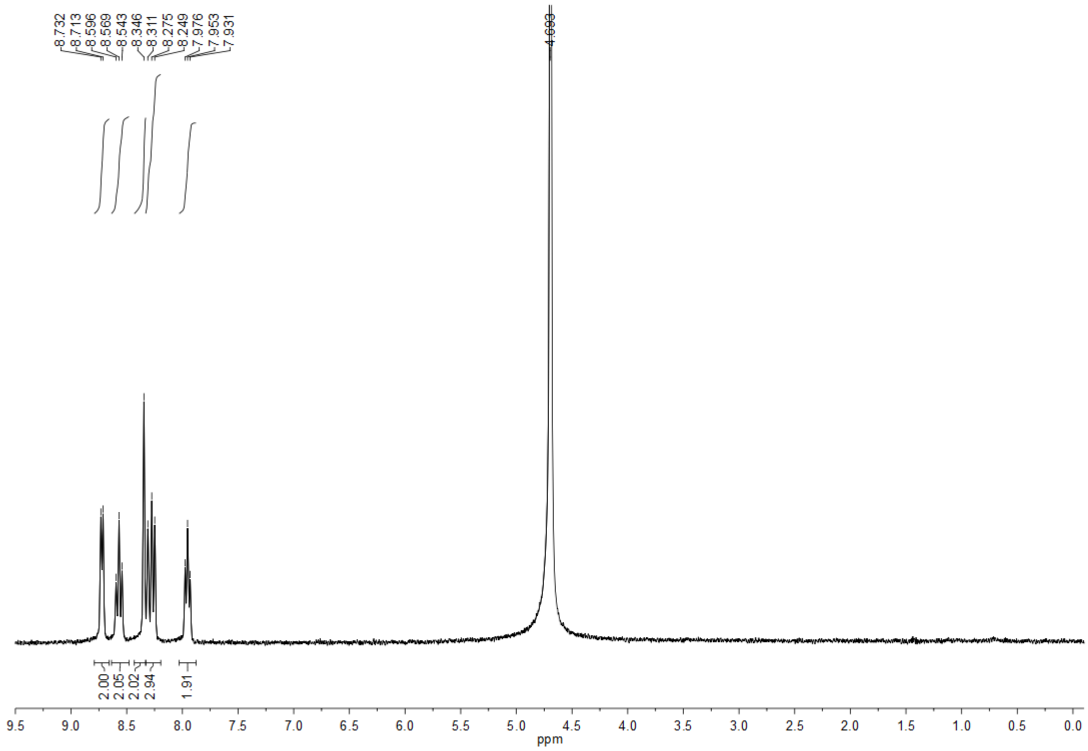

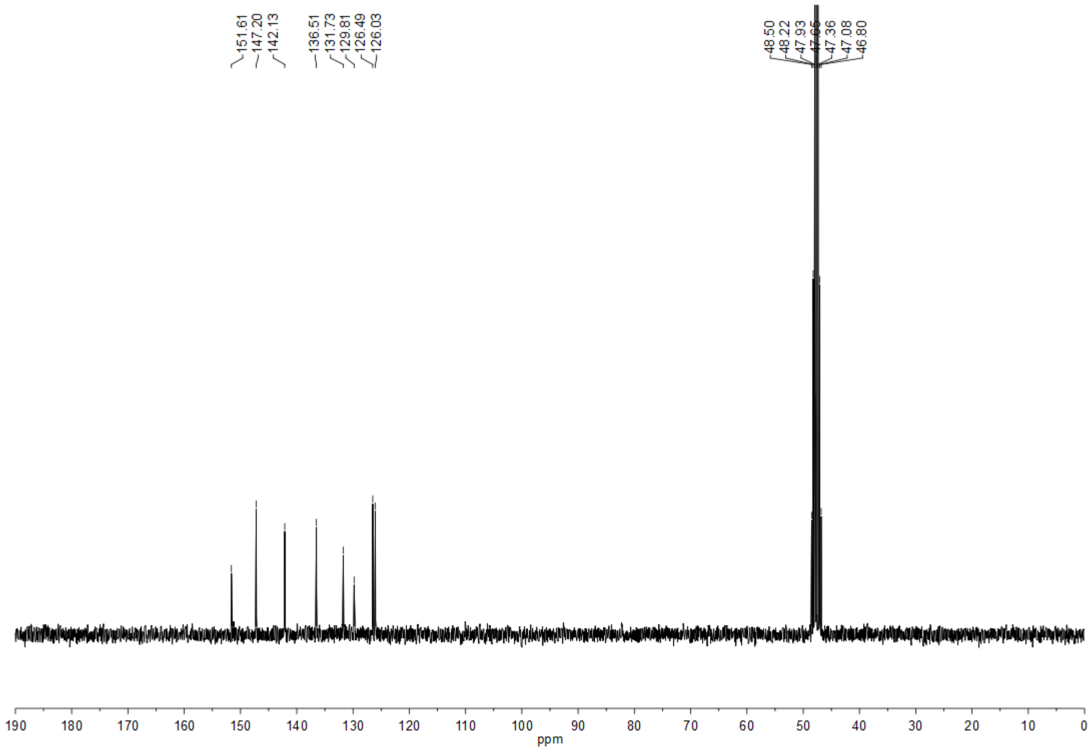

^{1}H NMR and ^{13}C NMR spectra of boronic acid **8**:

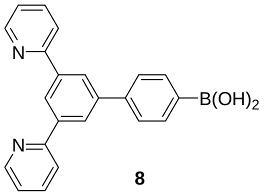

8

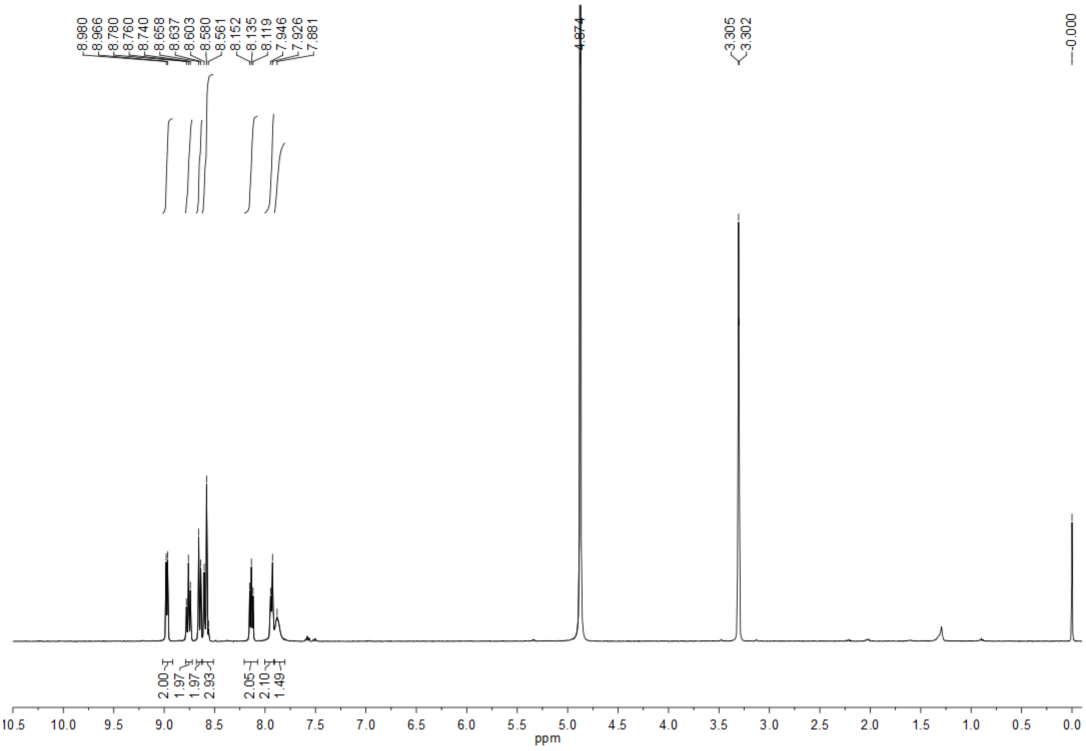

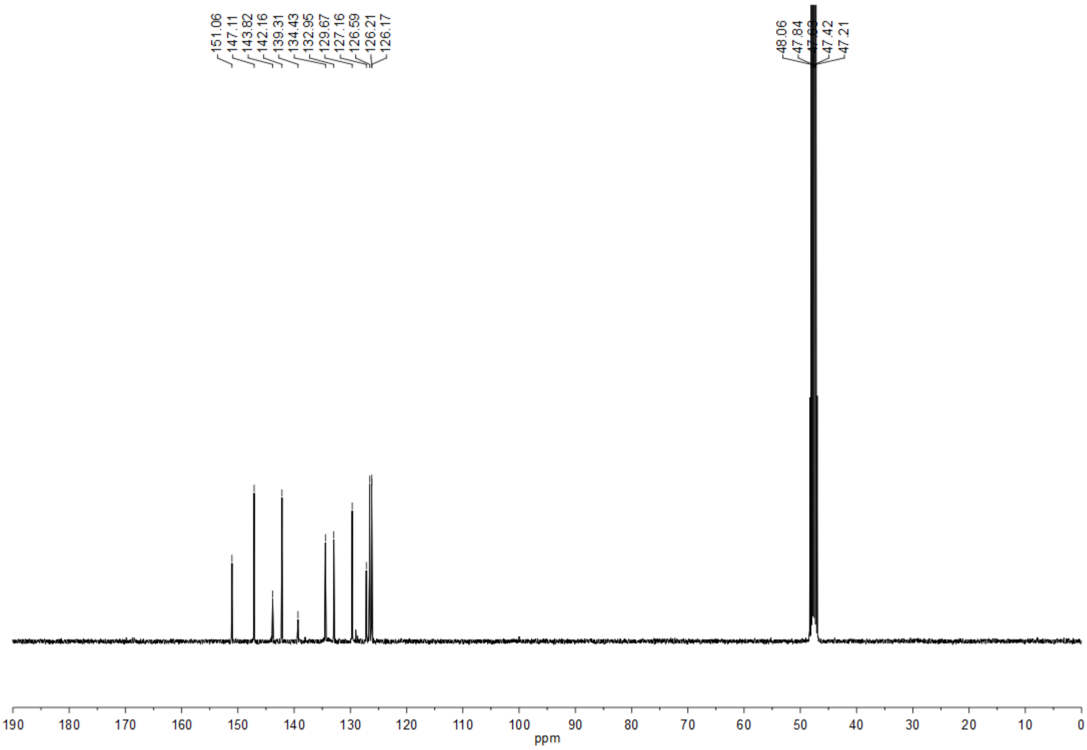

^{1}H NMR and ^{13}C NMR spectra of boronic acid **9**:

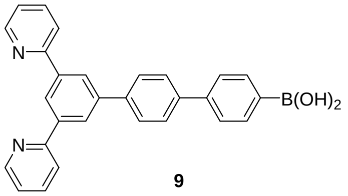

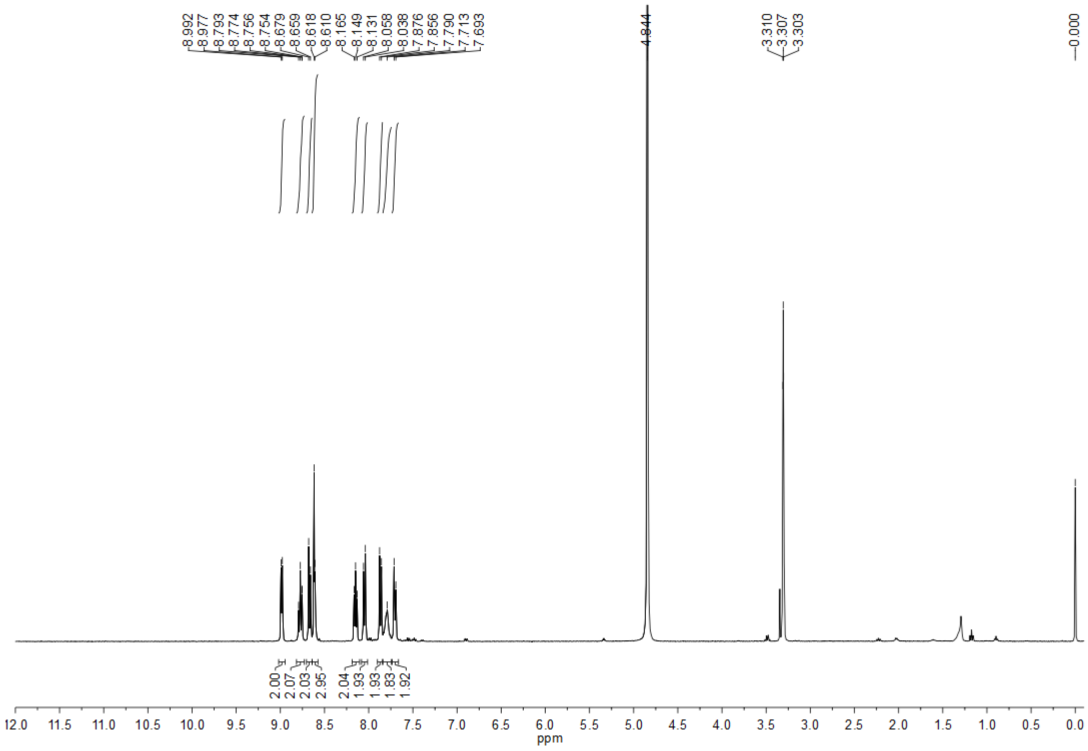

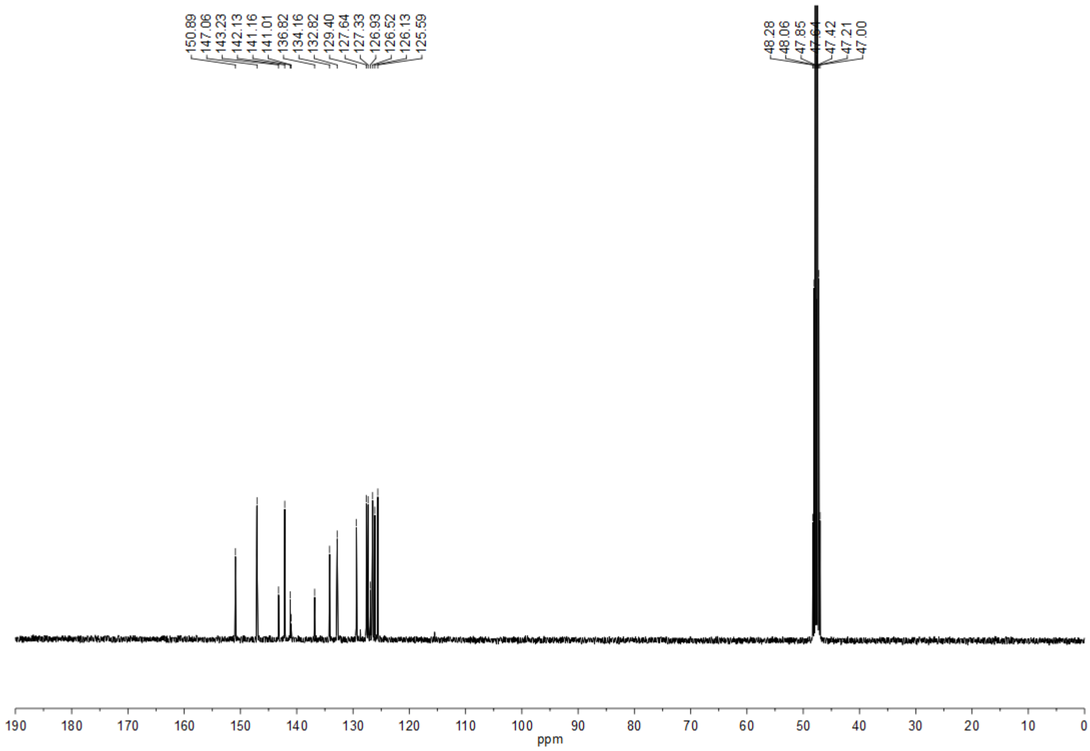

^{1}H NMR and ^{13}C NMR spectra of bromide **12**:

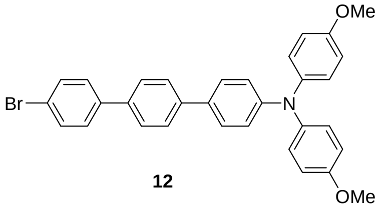

12

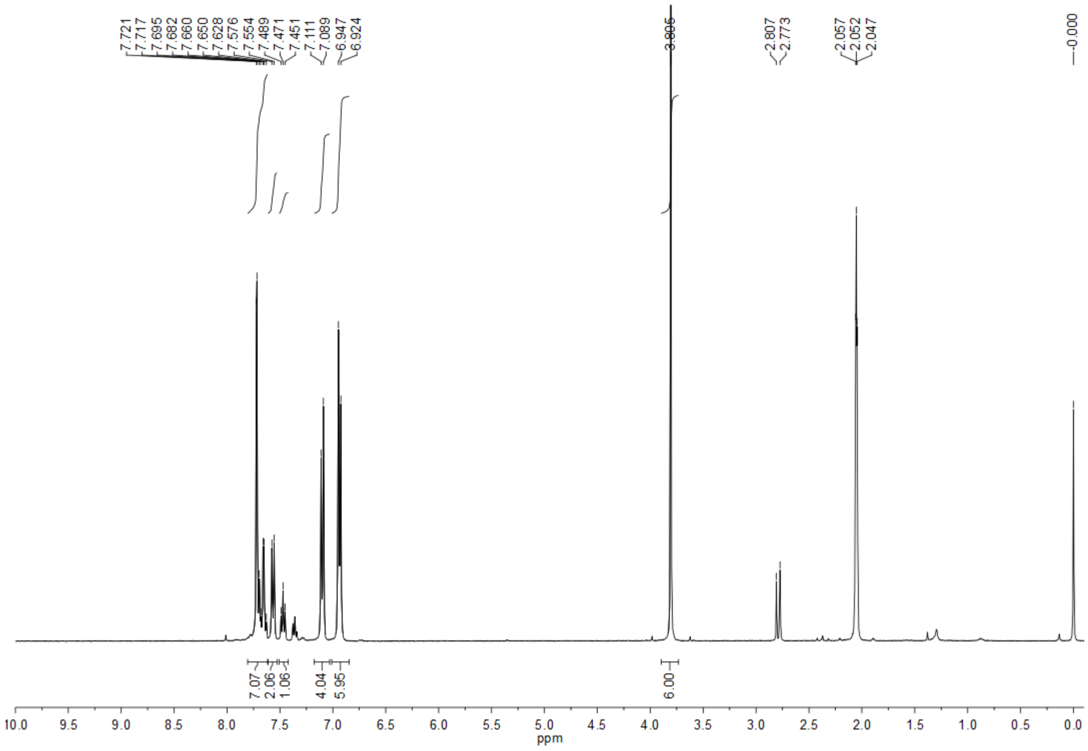

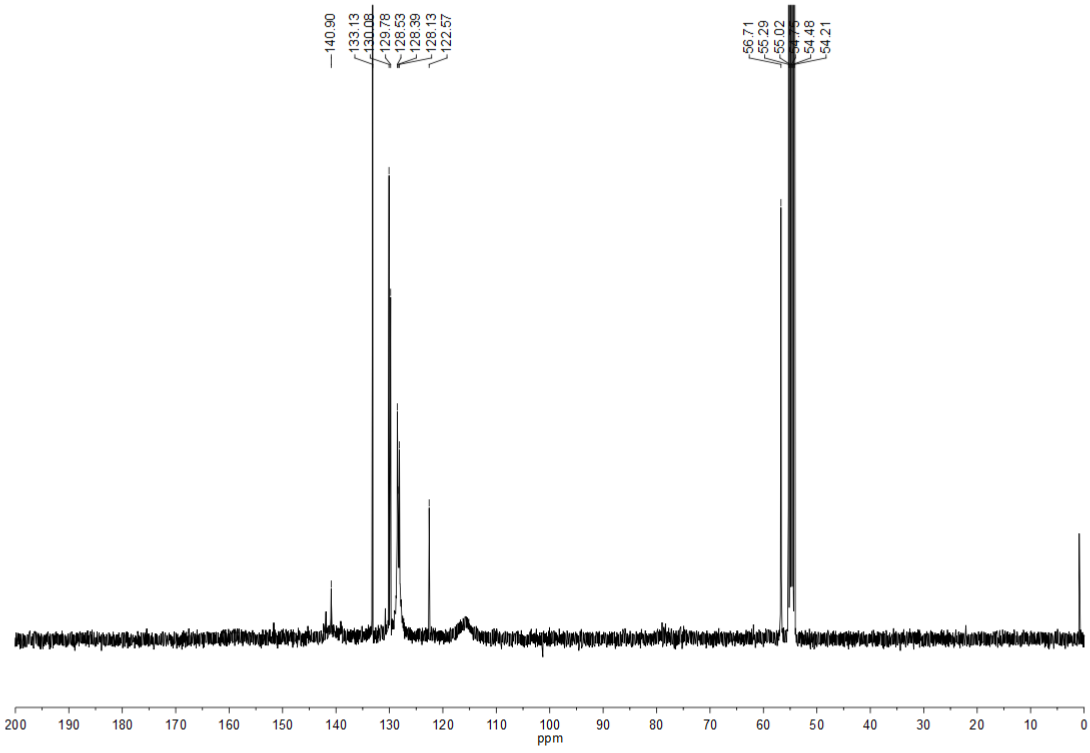

^{1}H NMR and ^{13}C NMR spectra of 1,3-di(pyrid-2-yl)-5-(*para*-bromophenyl)benzene:

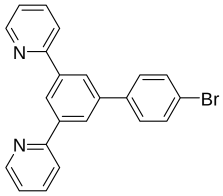

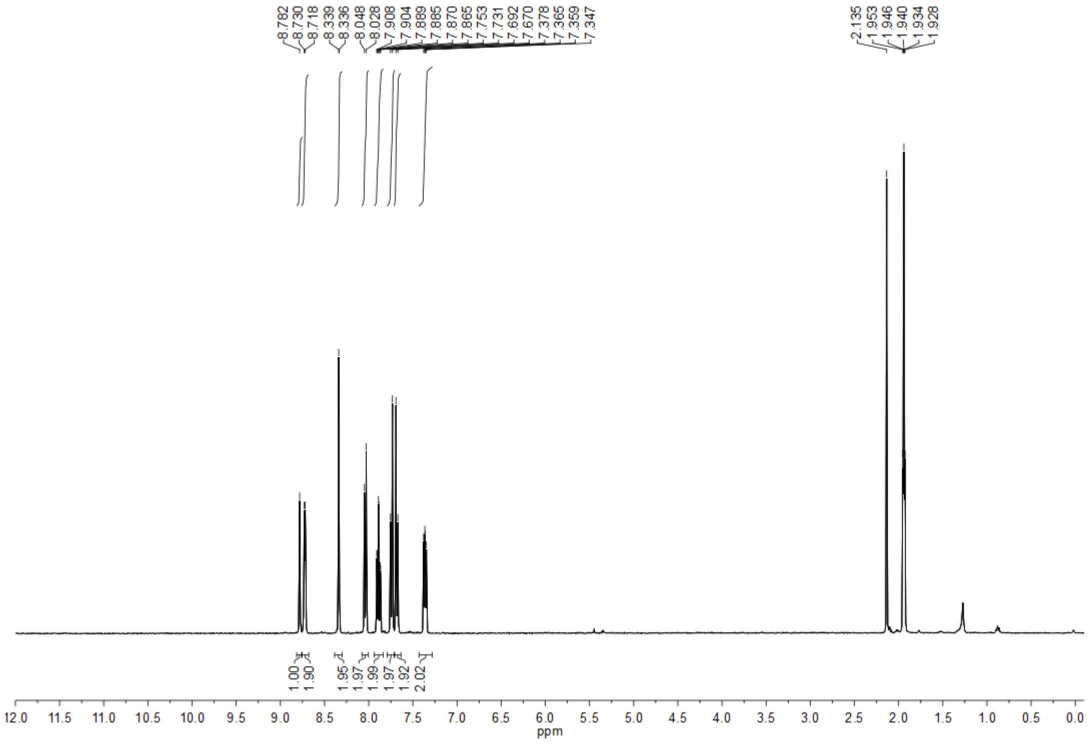

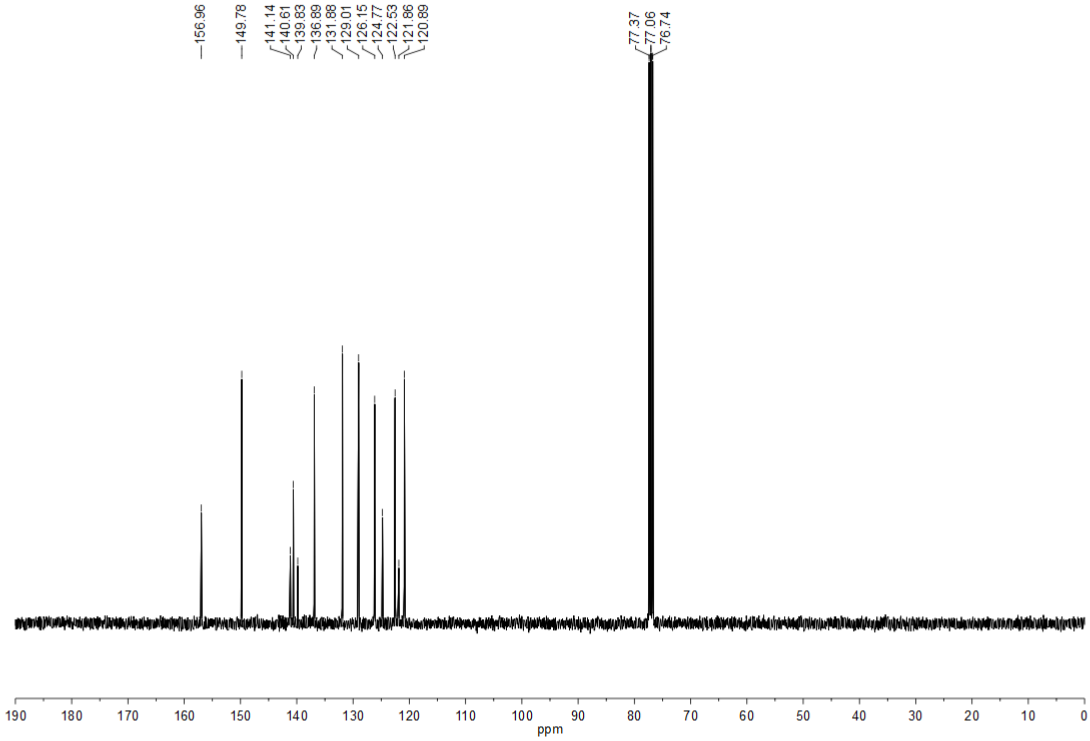

^{1}H NMR and ^{13}C NMR spectra of 1,3-di(pyrid-2-yl)-5-(4′′-bromobiphen-4′-yl)-benzene:

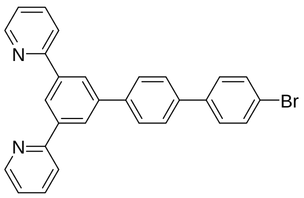

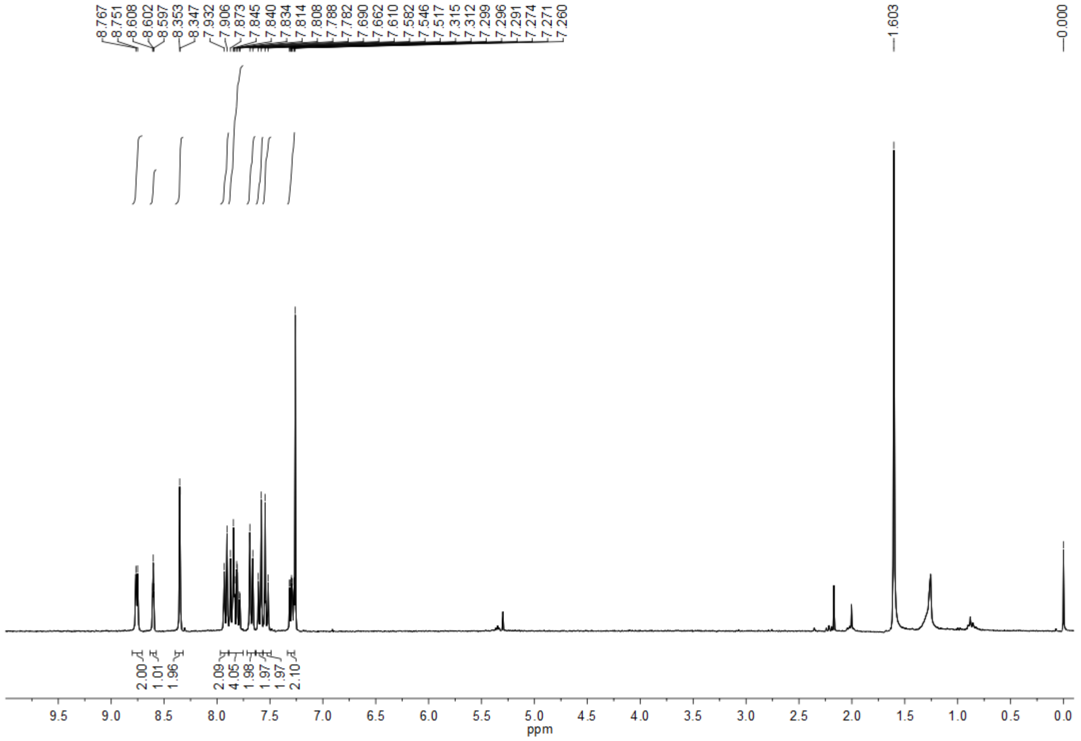

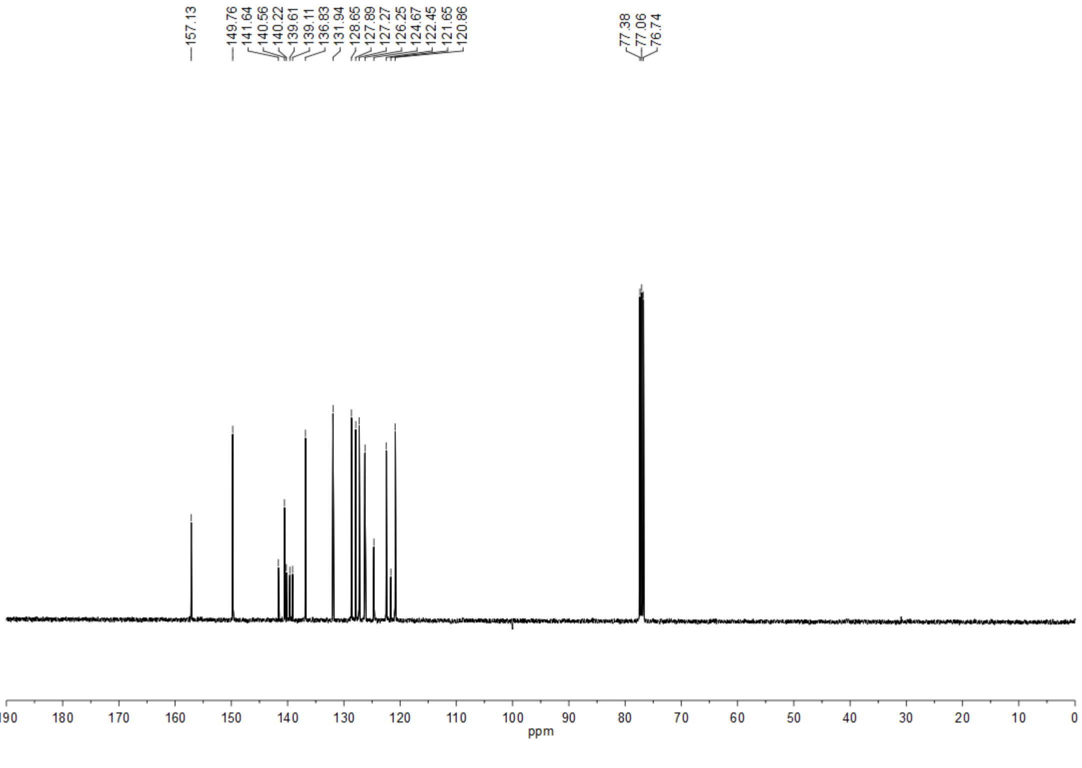

^{1}H NMR and ^{13}C NMR spectra of ligand **14**:

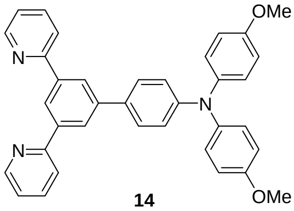

14

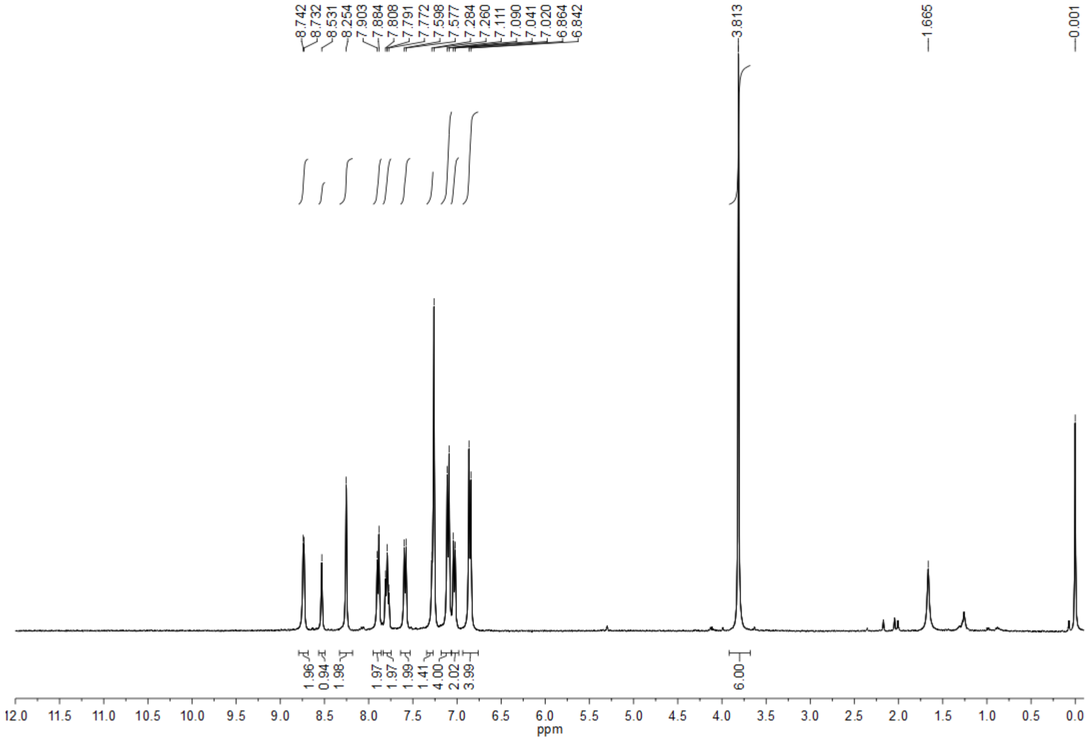

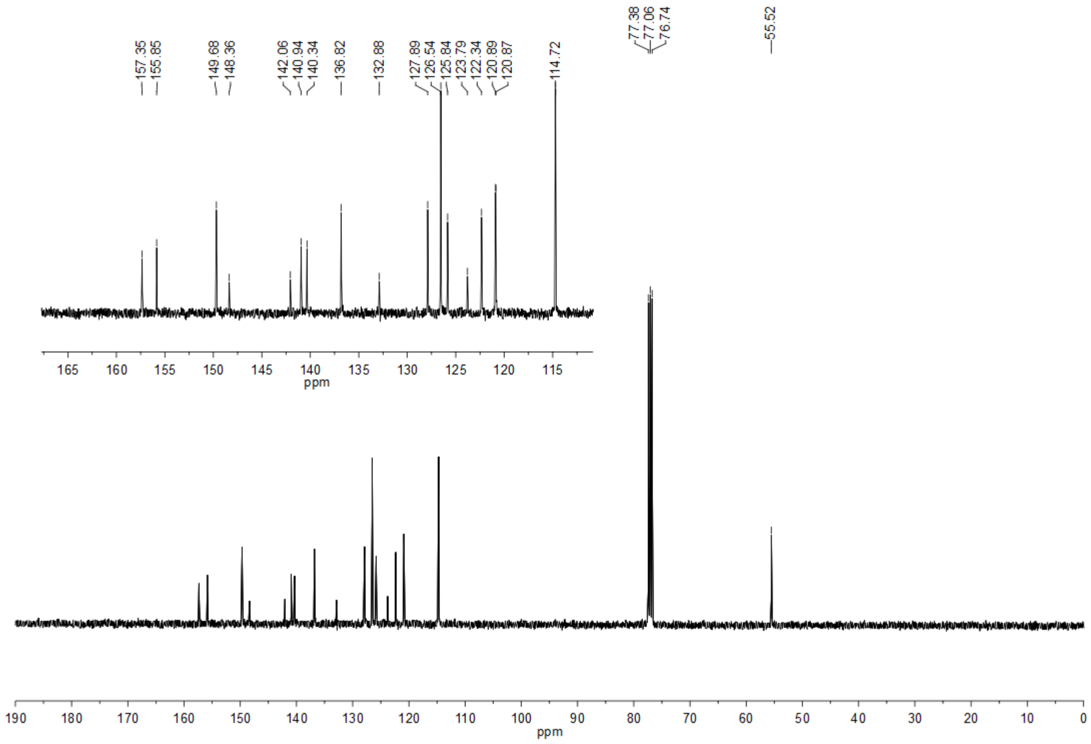

^{1}H NMR and ^{13}C NMR spectra of ligand **15**:

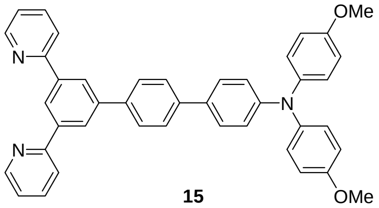

15

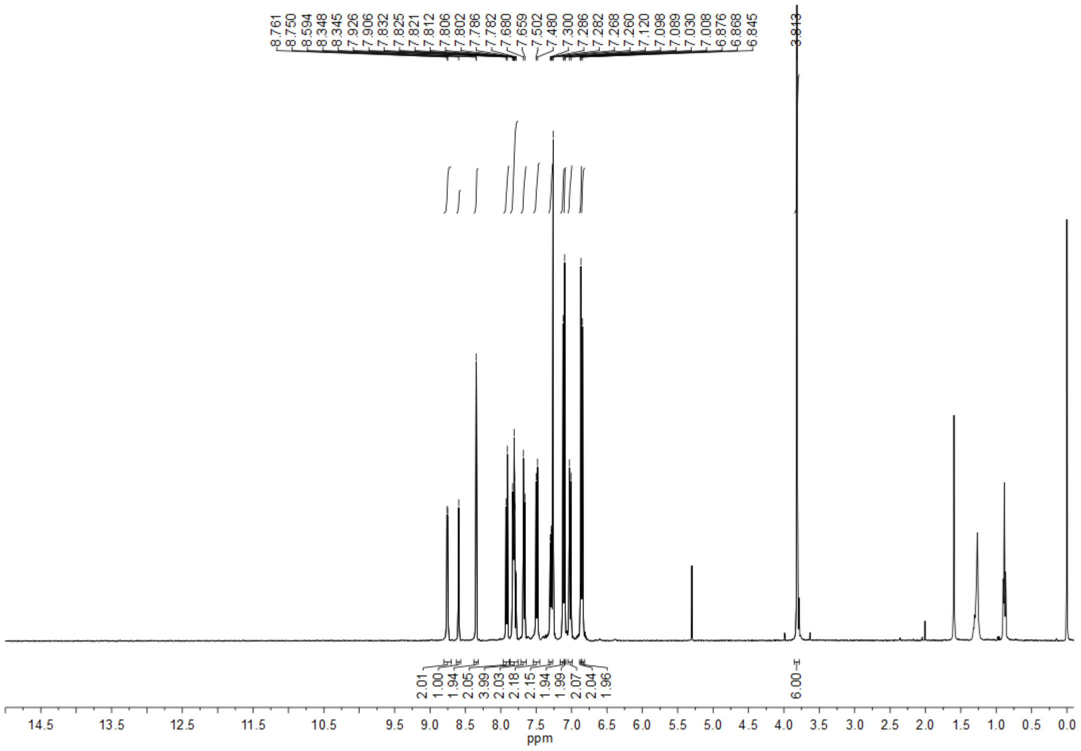

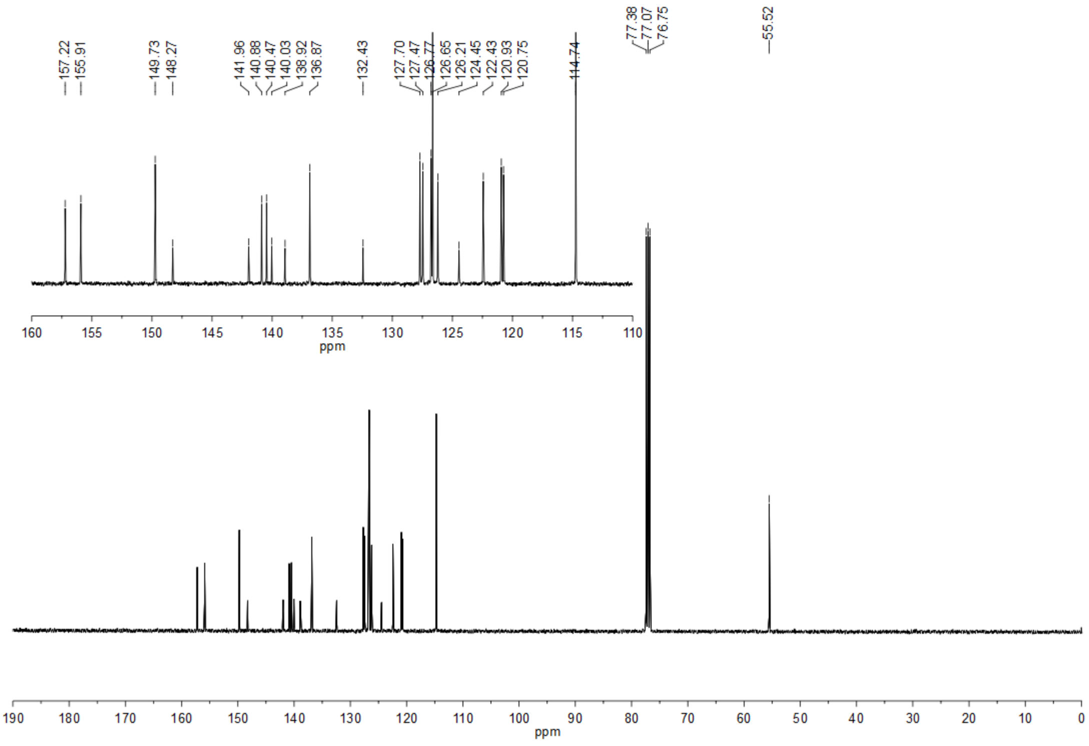

^{1}H NMR and ^{13}C NMR spectra of ligand **16**:

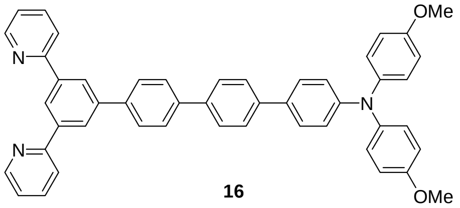

16

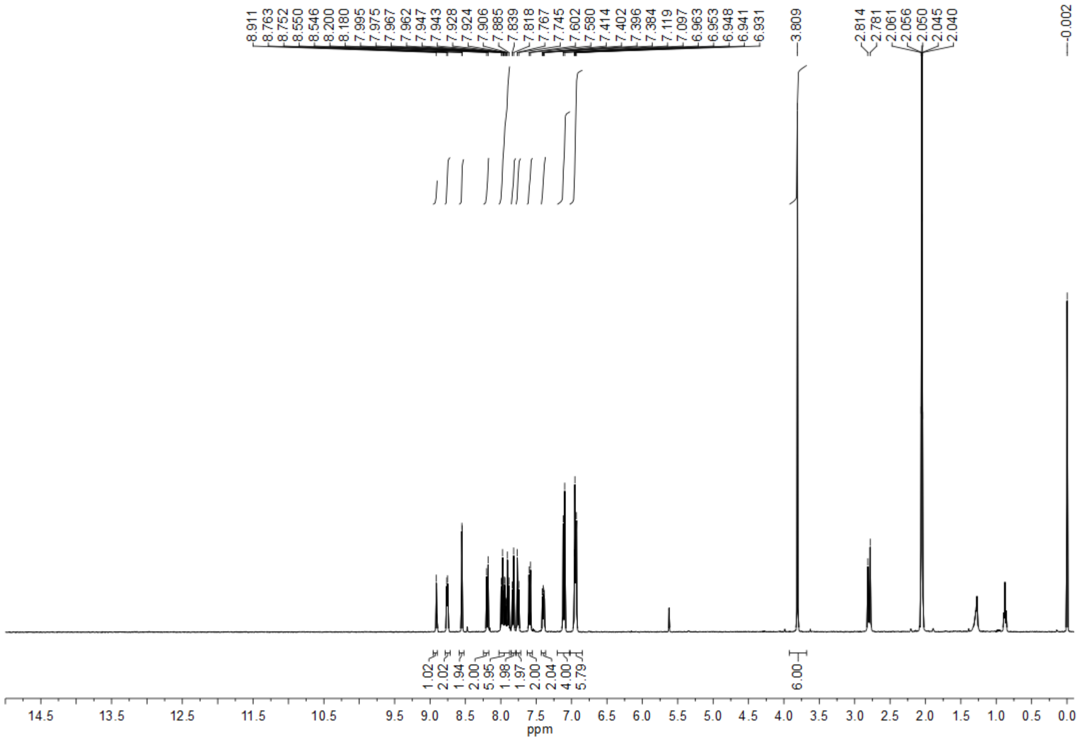

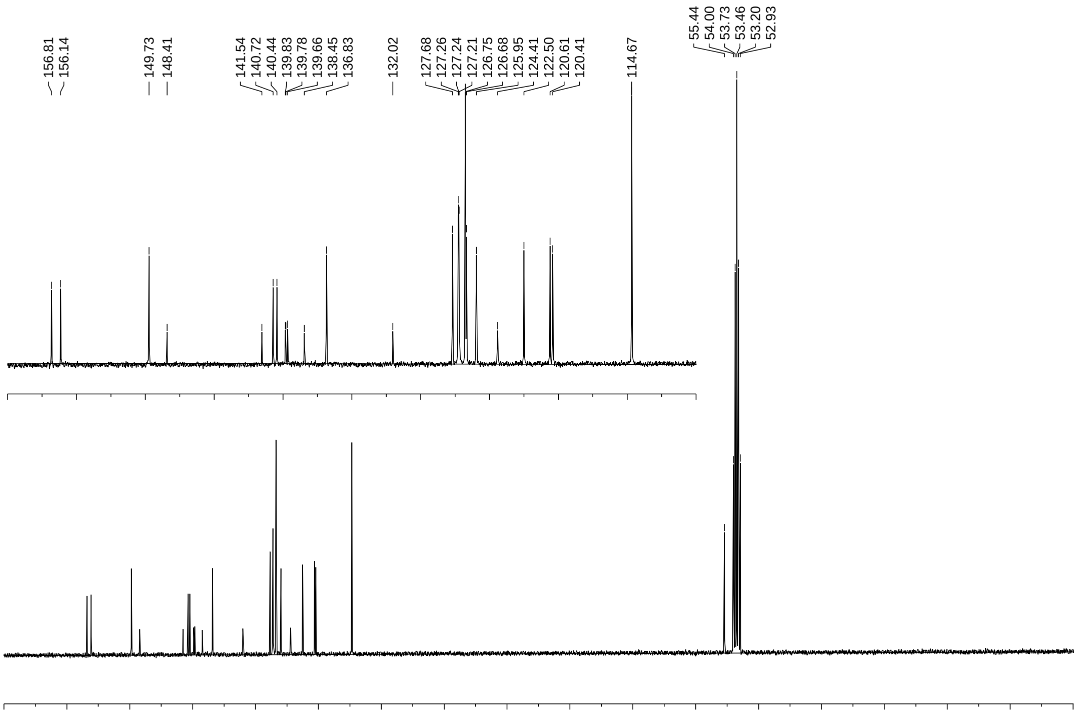

^{1}H NMR and ^{13}C NMR spectra of ligand **17**:

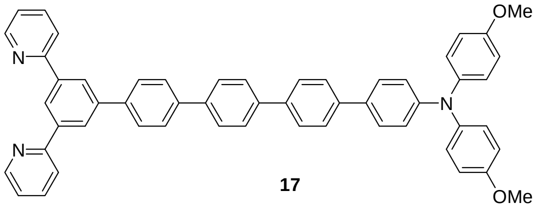

17

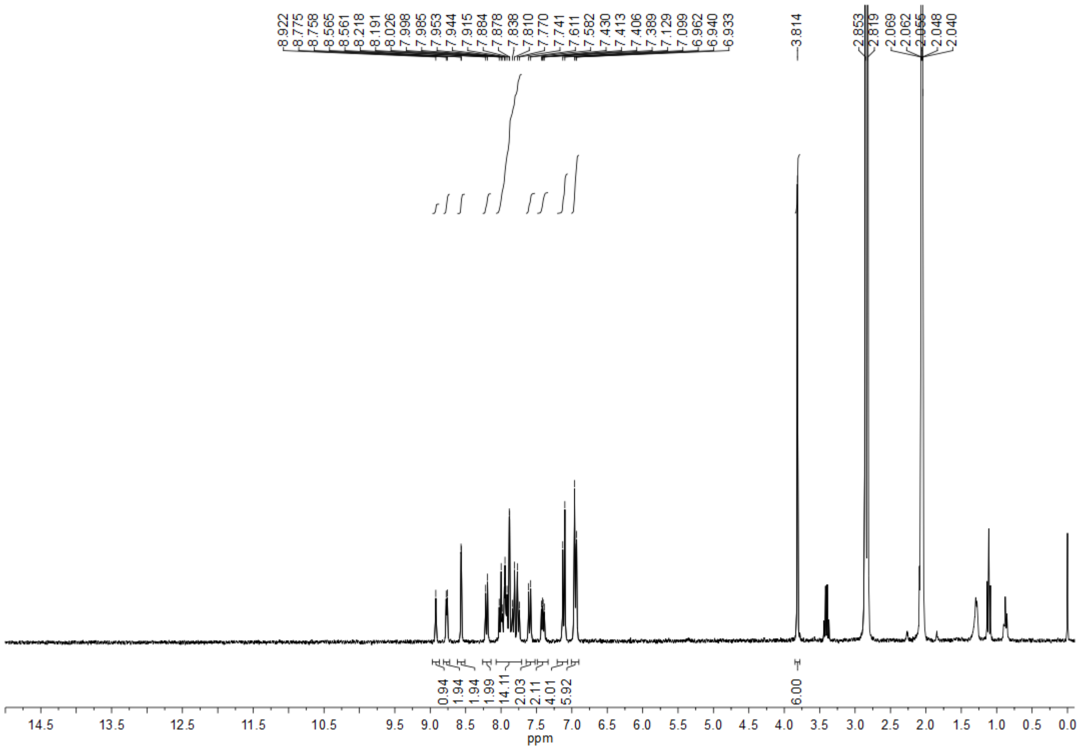

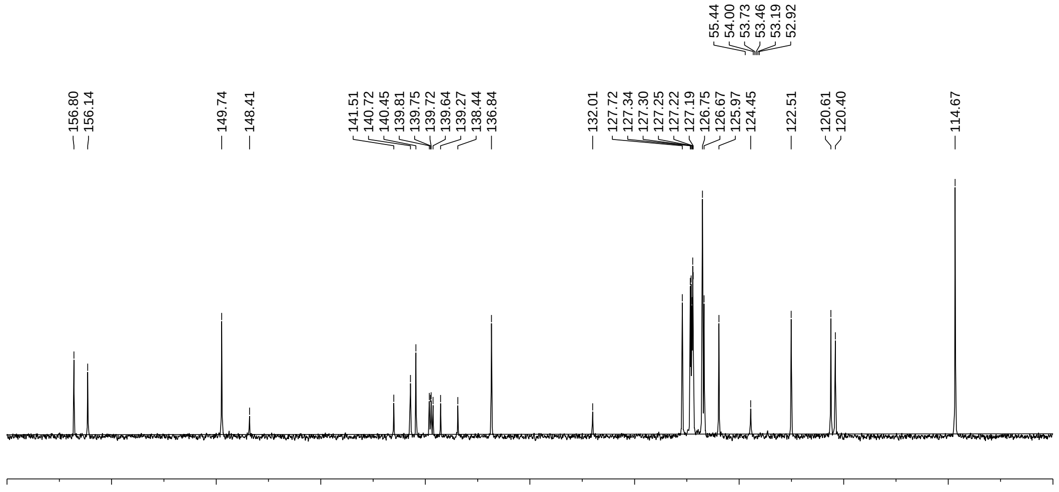

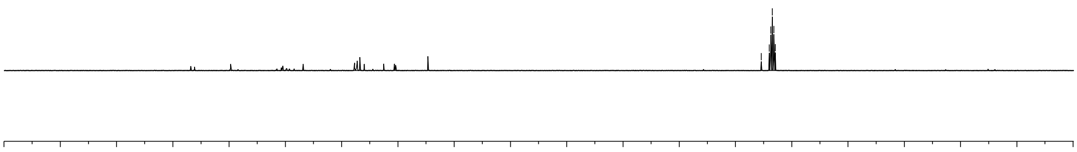

^{1}H NMR and ^{13}C NMR spectra of ligand **18**:

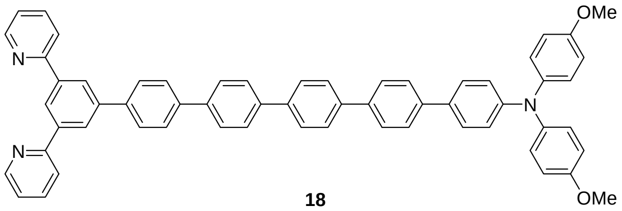

18

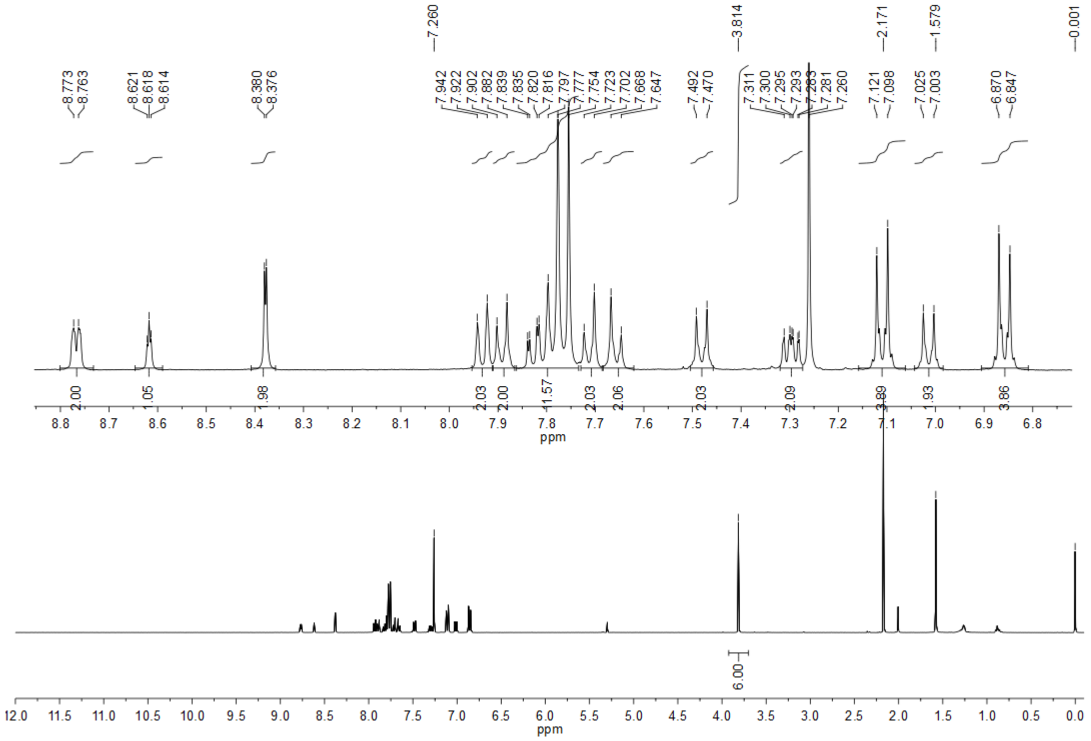

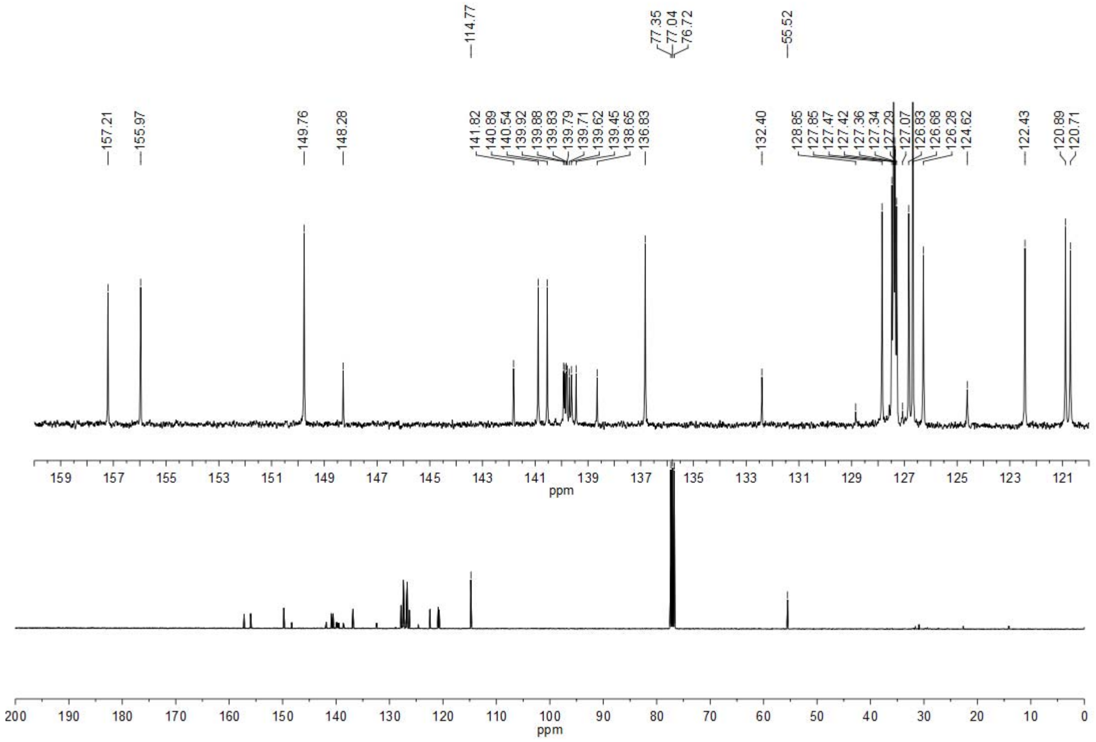

^{1}H NMR and MALDI-TOF mass spectra of complex **2**(PF_6):

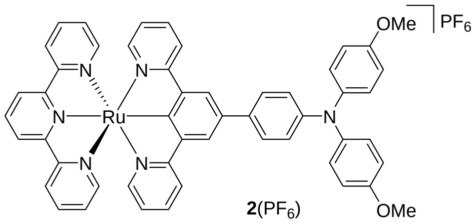

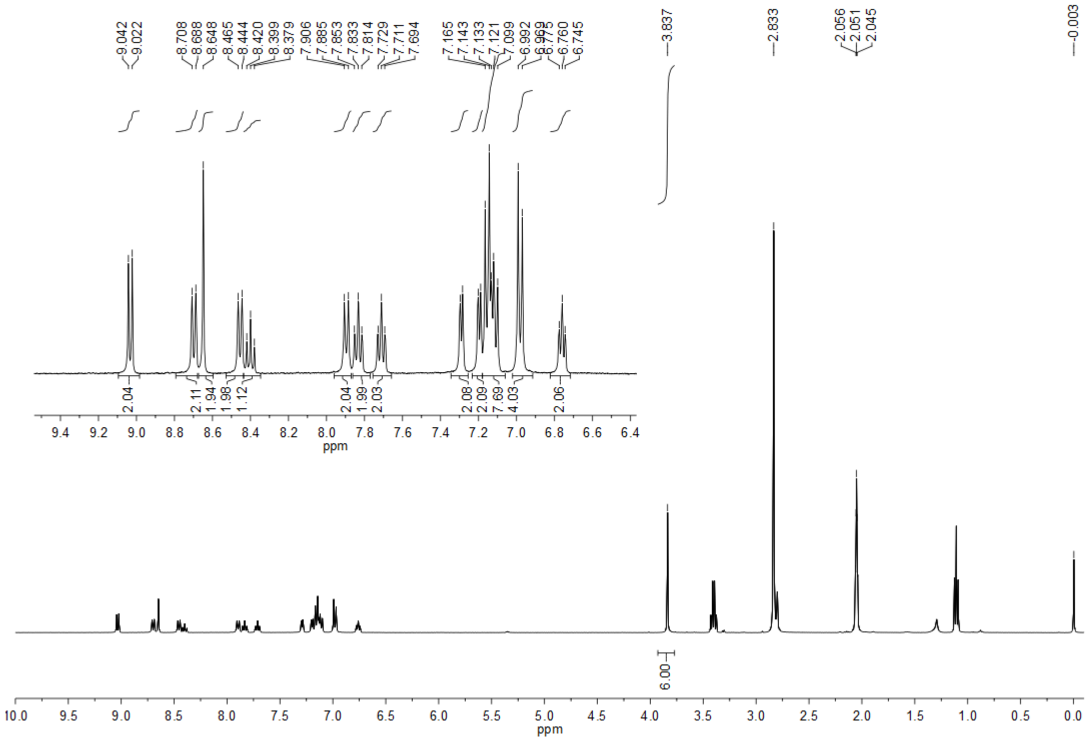

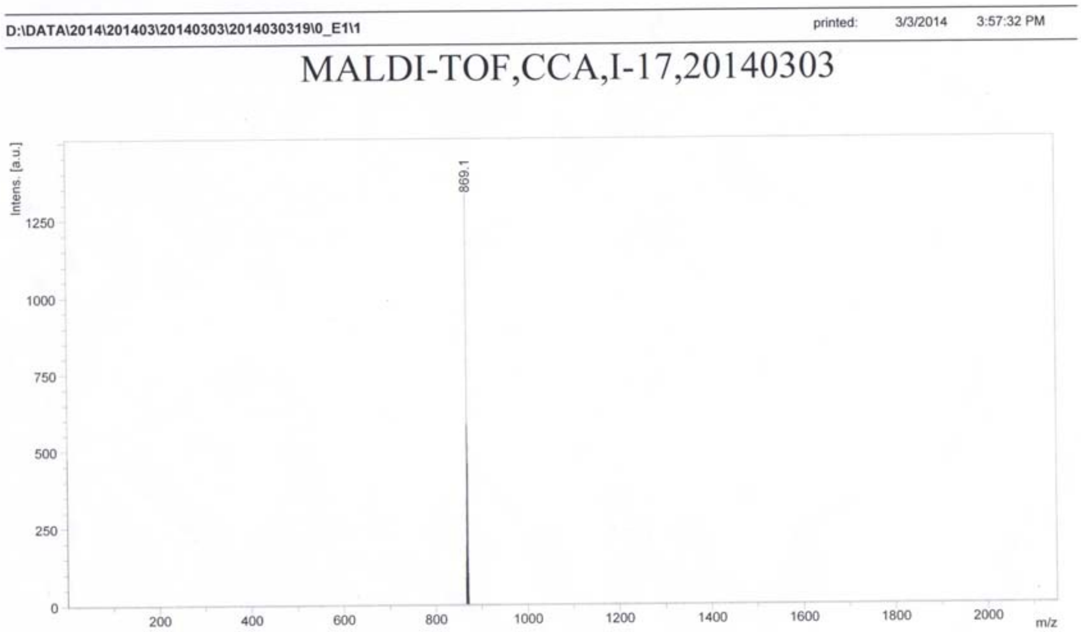

^{1}H NMR and MALDI-TOF mass spectra of complex **3**(PF_6):

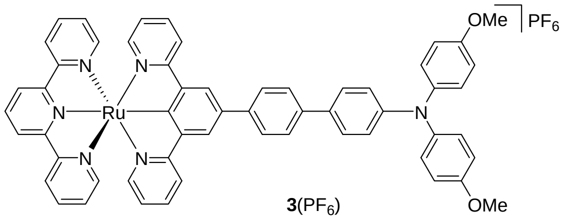

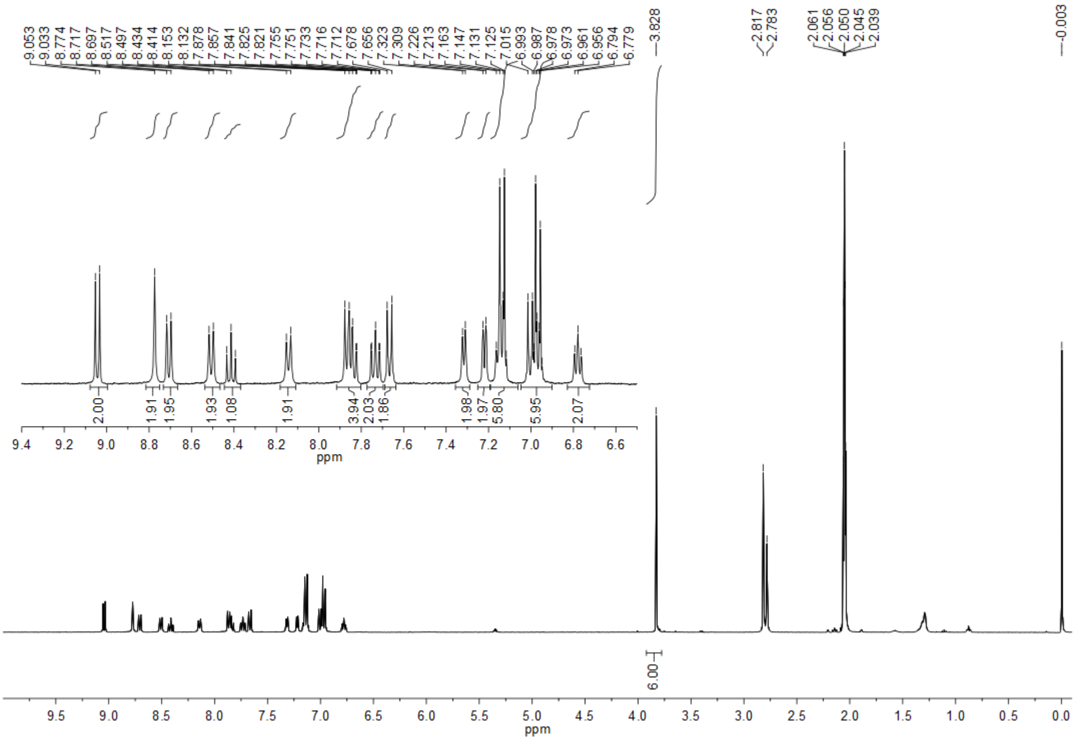

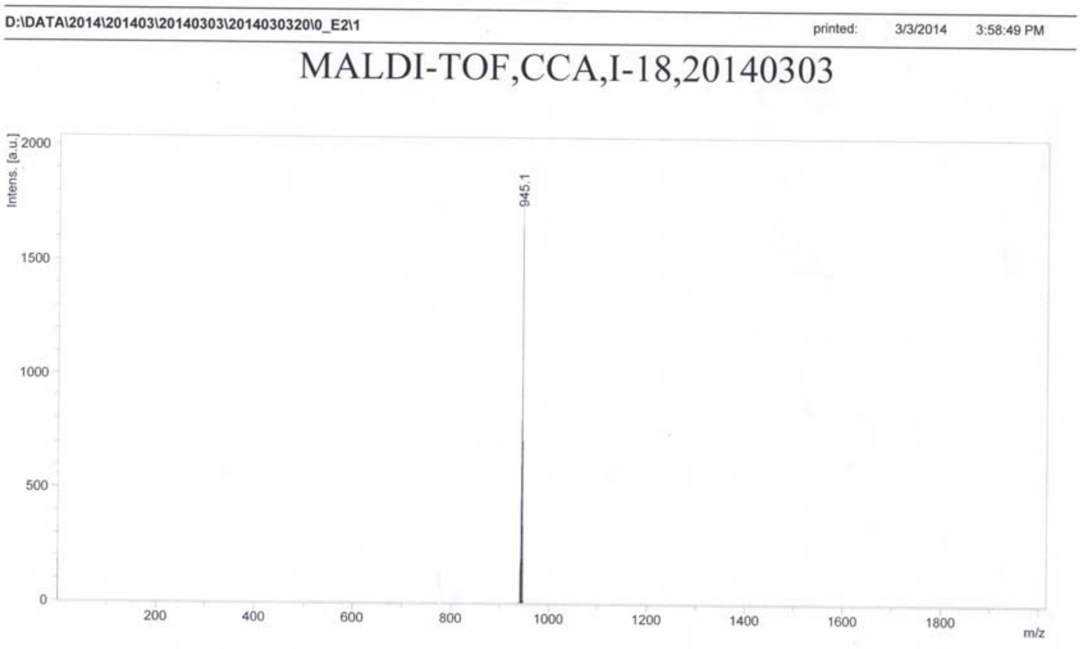

^{1}H NMR and MALDI-TOF mass spectra of complex **4**(PF_6):

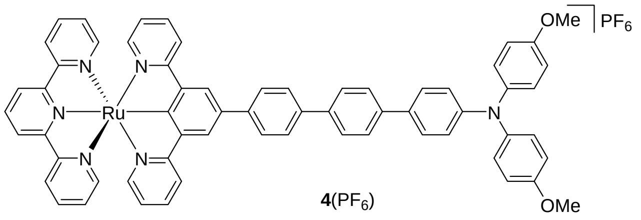

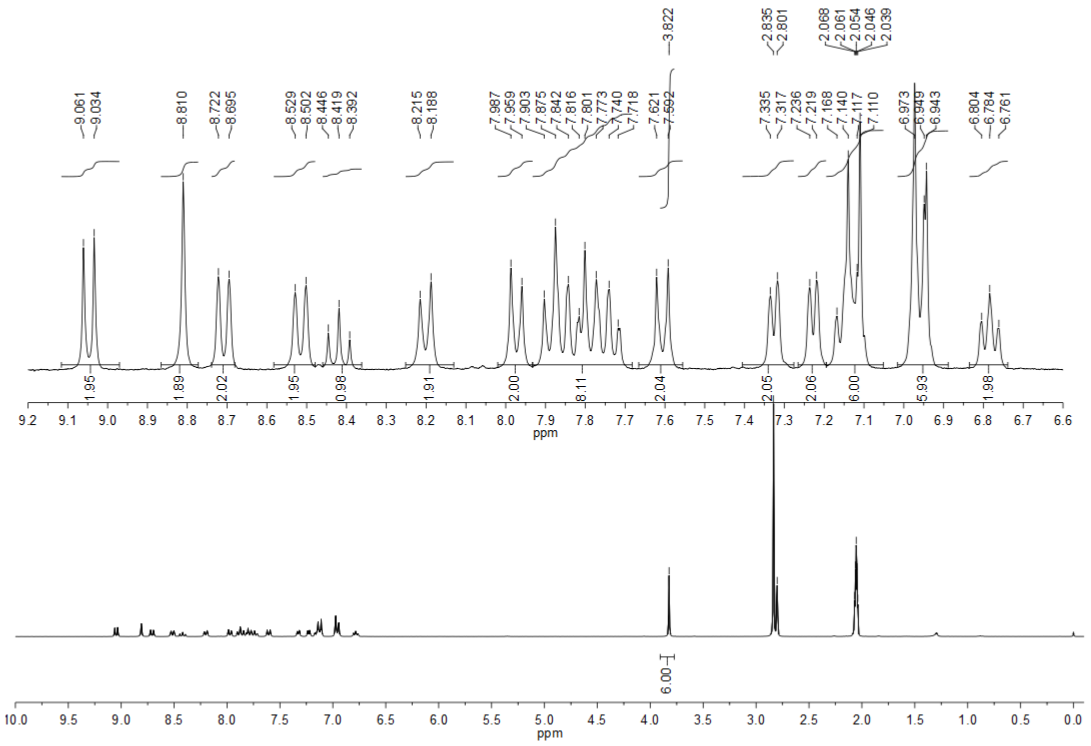

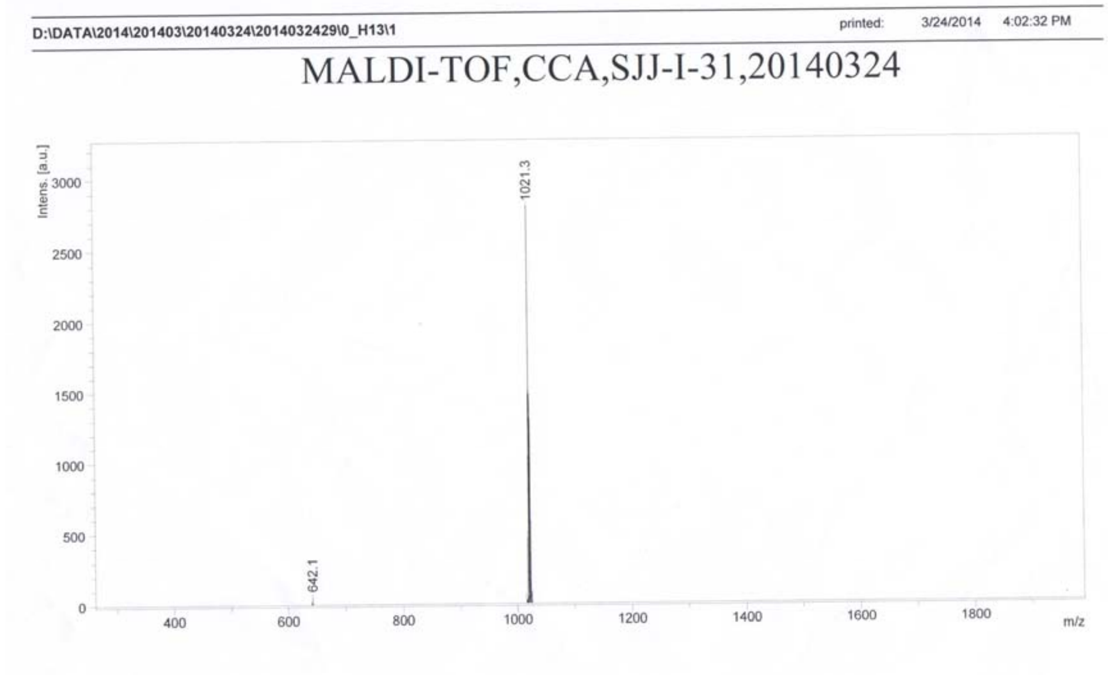

^{1}H NMR and MALDI-TOF mass spectra of complex **5**(PF_6):

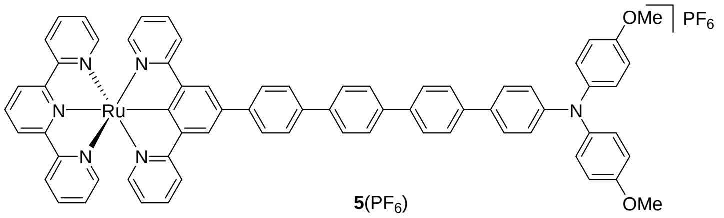

5(PF_6)

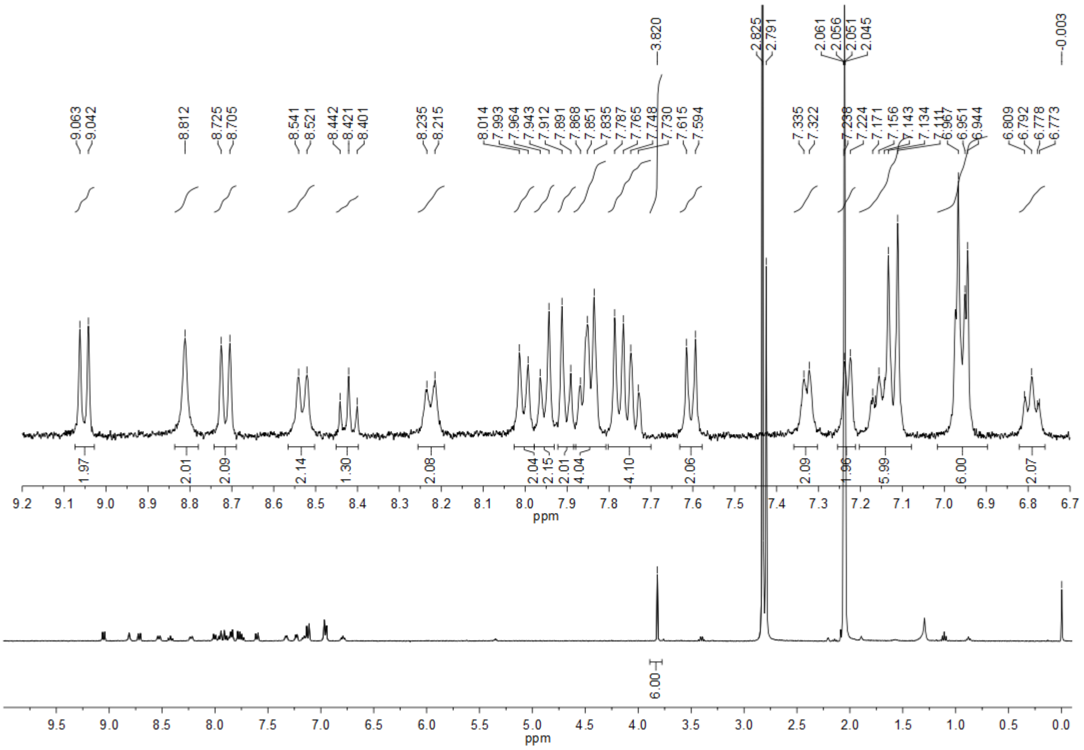

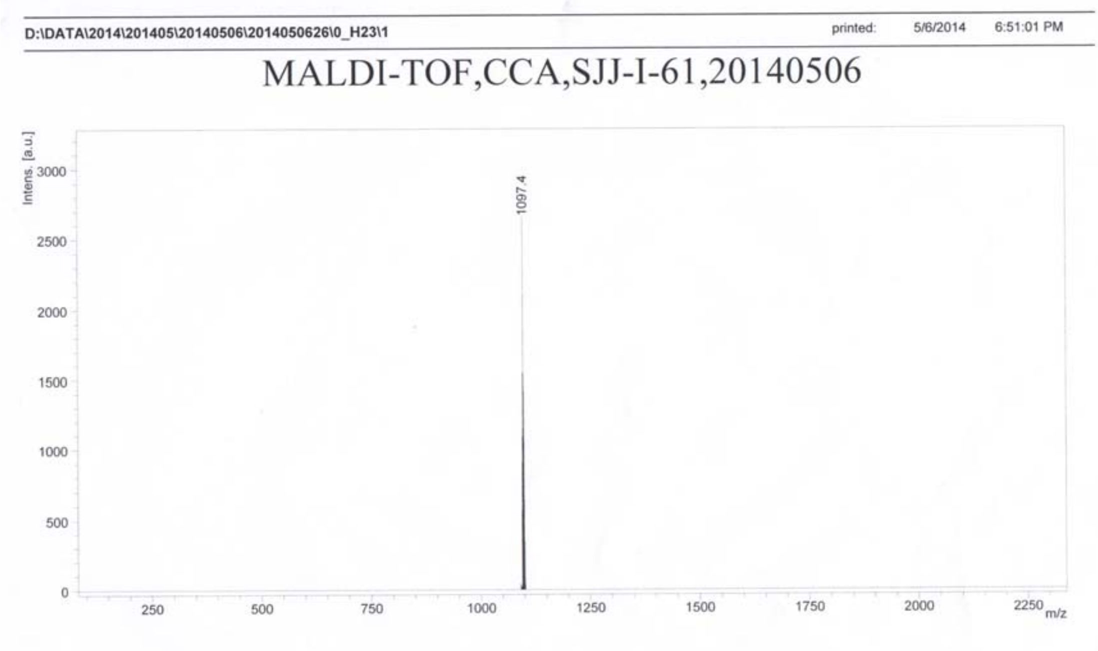

^{1}H NMR and MALDI-TOF mass spectra of complex **6**(PF_6):

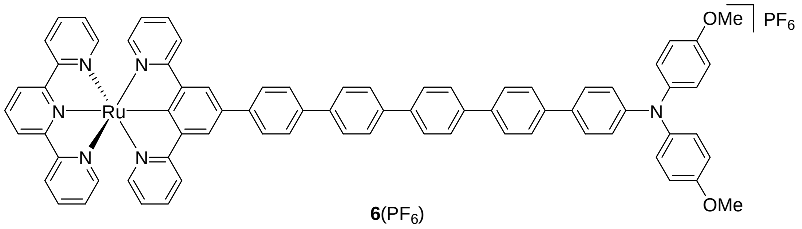

6(PF_6)

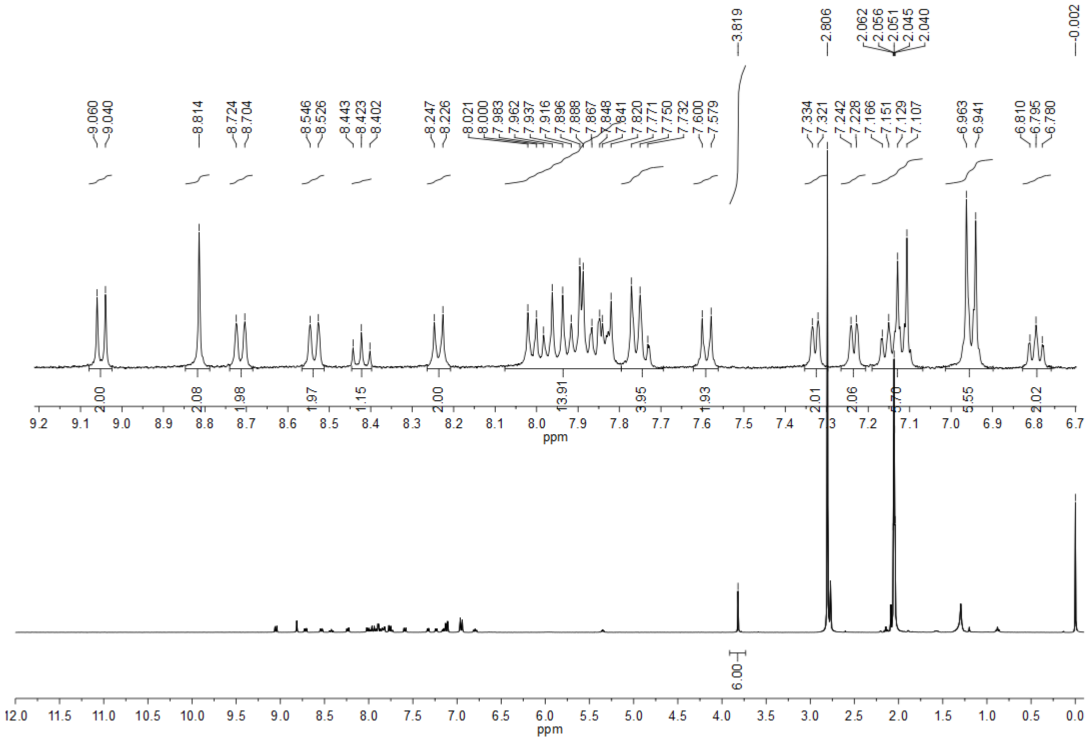

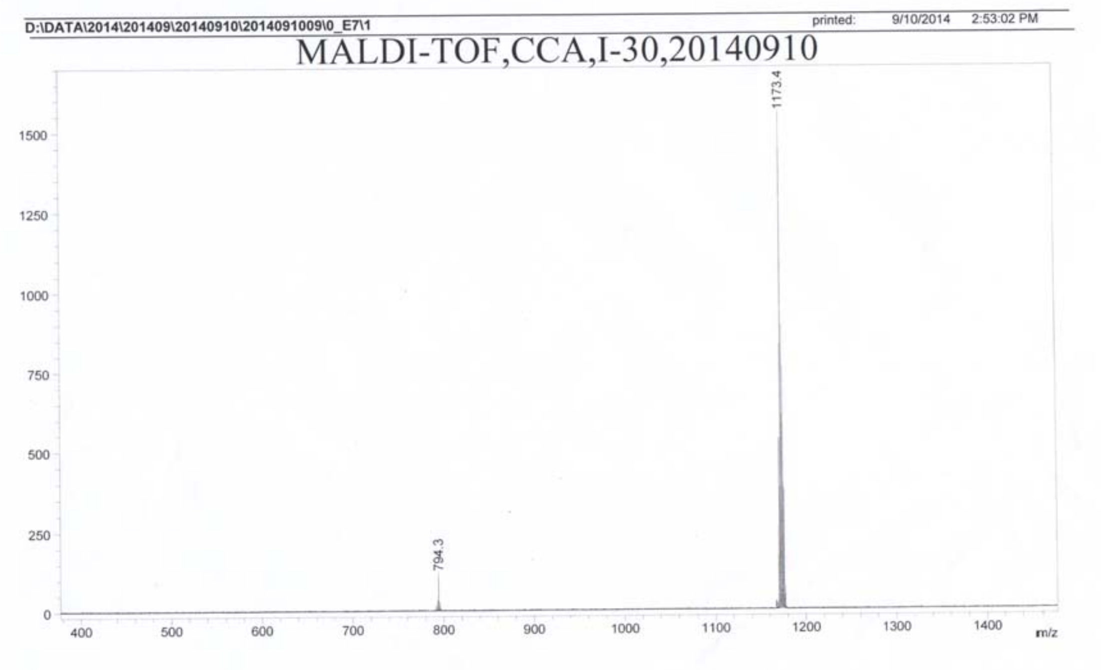

Supplement: Supporting information [file srep13835-s1.pdf]
